# A *Pseudomonas aeruginosa* type VI secretion system regulated by CueR facilitates copper acquisition

**DOI:** 10.1371/journal.ppat.1008198

**Published:** 2019-12-02

**Authors:** Yuying Han, Tietao Wang, Gukui Chen, Qinqin Pu, Qiong Liu, Yani Zhang, Linghui Xu, Min Wu, Haihua Liang

**Affiliations:** 1 Key Laboratory of Resources Biology and Biotechnology in Western China, Ministry of Education, College of Life Sciences, Northwest University, Xi'an, ShaanXi, China; 2 Department of Basic Science, School of Medicine and Health Science, University of North Dakota, Grand Forks, North Dakota, United States of America; 3 Guangdong Province Key Laboratory of Microbial Signals and Disease Control, Integrative Microbiology Research Centre, South China Agricultural University, Guangzhou, GuangDong, China; Channing Laboratory, Brigham and Women's Hospital, UNITED STATES

## Abstract

The type VI secretion system (T6SS) is widely distributed in Gram-negative bacteria, whose function is known to translocate substrates to eukaryotic and prokaryotic target cells to cause host damage or as a weapon for interbacterial competition. *Pseudomonas aeruginosa* encodes three distinct T6SS clusters (H1-, H2-, and H3-T6SS). The H1-T6SS-dependent substrates have been identified and well characterized; however, only limited information is available for the H2- and H3-T6SSs since relatively fewer substrates for them have yet been established. Here, we obtained *P*. *aeruginosa* H2-T6SS-dependent secretomes and further characterized the H2-T6SS-dependent copper (Cu^2+^)-binding effector azurin (Azu). Our data showed that both *azu* and H2-T6SS were repressed by CueR and were induced by low concentrations of Cu^2+^. We also identified the Azu-interacting partner OprC, a Cu^2+^-specific TonB-dependent outer membrane transporter. Similar to H2-T6SS genes and *azu*, expression of *oprC* was directly regulated by CueR and was induced by low Cu^2+^. In addition, the Azu-OprC-mediated Cu^2+^ transport system is critical for *P*. *aeruginosa* cells in bacterial competition and virulence. Our findings provide insights for understanding the diverse functions of T6SSs and the role of metal ions for *P*. *aeruginosa* in bacteria-bacteria competition.

## Author summary

The type VI secretion system (T6SS) is a specific macromolecular protein export apparatus, and widely distributed in Gram-negative bacteria. T6SS plays an important role in anti-bacterial competition or delivers effector proteins to both eukaryotic and prokaryotic cells. In the present study, we performed secretomes analysis and identified 21 substrates of *P*. *aeruginosa* H2-T6SS-dependent. Specifically, we report a Cu^2+^-scavenging pathway consisting of a copper transporter, OprC, and a type VI secretion system (H2-T6SS)-secreted Cu^2+^-binding protein, Azu. Both of them are under control of the transcriptional regulator CueR. Indeed, the Azu-OprC-mediated Cu^2+^ transport system is critical for *P*. *aeruginosa* cells in bacterial competition and virulence. These findings exemplify how *P*. *aeruginosa* deploys this metal system to adapt to the complex environment during evolution.

## Introduction

Copper (Cu) is an indispensable metal ion that plays a crucial role in the development of almost all aspects of mammalian physiology; therefore, defects in Cu homeostasis impact everything from immune responses to microbial infection [1, 2]. Cu can undergo reversible oxidation states between reduced Cu^+^ and oxidized Cu^2+^ and has a high redox potential, making it a critical cofactor of enzymes used for electron transfer reactions in the presence of oxygen [2, 3]. Given the essential role of Cu in bacterial physiology, it is not surprising that restriction of this micronutrient is an important innate defense strategy [4]. Therefore, organisms exert tight control over Cu^2+^ transport and traffic through different compartments, ensuring the homeostasis required for cuproprotein synthesis and prevention of toxic effects [2]. A number of cytoplasmic Cu^2+^-sensing transcriptional regulators (CueR, CsoR, CopY) [5–7], as well as periplasmic Cu^2+^-sensing two-component systems (CopR/S, CusR/S, PcoR/S) [3, 8], have been identified for the import of Cu. The transmembrane P_1B_-type ATPases are also responsible for cytoplasmic Cu^2+^ efflux [9]. Azurin (Azu) is a high affinity of oxidized copper (Cu^2+^)-bound protein [10], which coordinates the activity of respiratory metalloenzymes as cofactors by copper uptake in *Pseudomonas aeruginosa* [11]. Recently, Quintana *et al* have established a system-wide model of a Cu homeostasis network in *P*. *aeruginosa* [12]. Although insights into the mechanisms of metal regulation and transport have been achieved, little is known on the importance of Cu^2+^ availability in bacterial pathogenesis.

The type VI secretion system (T6SS) is a widely distributed protein translocation apparatus used by many bacteria to deliver effector proteins to both eukaryotic and prokaryotic cells, which appear to be the major targets [13–16]. The components of T6SS have 14 core conserved genes, including TssA-TssM, Hcp, VgrG and ClpV, which form the key structures of T6SS [17]. Among them, VgrG forms a cell puncturing tip, and Hcp forms a tail-tube structure for effectors deliver. ClpV is a member of the AAA+ protein family [18]. ClpV and TssM are predicted to function as energizing components that are crucial for the T6SS secretion [13]. The T6SS is a versatile secretion system capable of performing diverse physiological functions, including biofilm formation, interbacterial interactions, acute and chronic infections, and stress response [14, 19, 20].

*P*. *aeruginosa* is an opportunistic Gram-negative pathogen distributed widely in the environment. It encodes several secretion systems described so far, including the T6SS. In its genome, three T6SS loci have been identified: the H1-, H2-, and H3-T6SSs [18]. The T6SS has been well-characterized to function in bacterial community competition by delivering toxins to target cells. For example, the Tse1-Tse3 proteins are H1-T6SS-dependent antibacterial toxin effectors that provide a fitness advantage during interbacterial competition [15]. Effectors PldA and TplE (Tle4) are both H2-T6SS substrates and exhibit antibacterial activity [16, 21]. The H2-T6SS-dependent substrate VgrG2b has been demonstrated as an anti-eukaryotic effector [22]. The H3-T6SS-dependent effector PldB targets the periplasm of prokaryotic cells and exerts antibacterial activity [23]. Bacteria are protected from self-intoxication by expression of an immunity protein, which inactivates its cognate effector [14, 23]. In addition to secretion of toxins, a recent report showed that the H3-T6SS is involved in iron acquisition in *P*. *aeruginosa* [24]. However, whether the H1- and H2-T6SSs mediated metal ions uptake remain unknown.

Although regulators of H2-T6SS expression have been characterized, a comprehensive secretome analysis has yet to be performed. Here, we used mass spectrometry (MS) to compare proteins in the secretomes of the Δ*retS* and Δ*retS*Δ*clpV2* strains. The secretome analysis revealed that the secretion of 21 proteins is dependent on the H2-T6SS, including the Cu^2+^-binding protein azurin (Azu). We further demonstrated that the H2-T6SS is involved in secretion of the effector Azu that helps to mediate Cu^2+^ acquisition. Uptake of Azu-bound Cu^2+^ required an outer membrane transporter, OprC, for active transport across the outer membrane under Cu^2+^-limited conditions. Cu acquisition mediated by the T6SS provides a growth advantage in bacterial competition and virulence. These findings expand our understanding of the diverse physiological functions of the T6SS and metal ion transport in bacteria.

## Results

### RetS negatively regulates H2-T6SS expression

**Fig 1:**
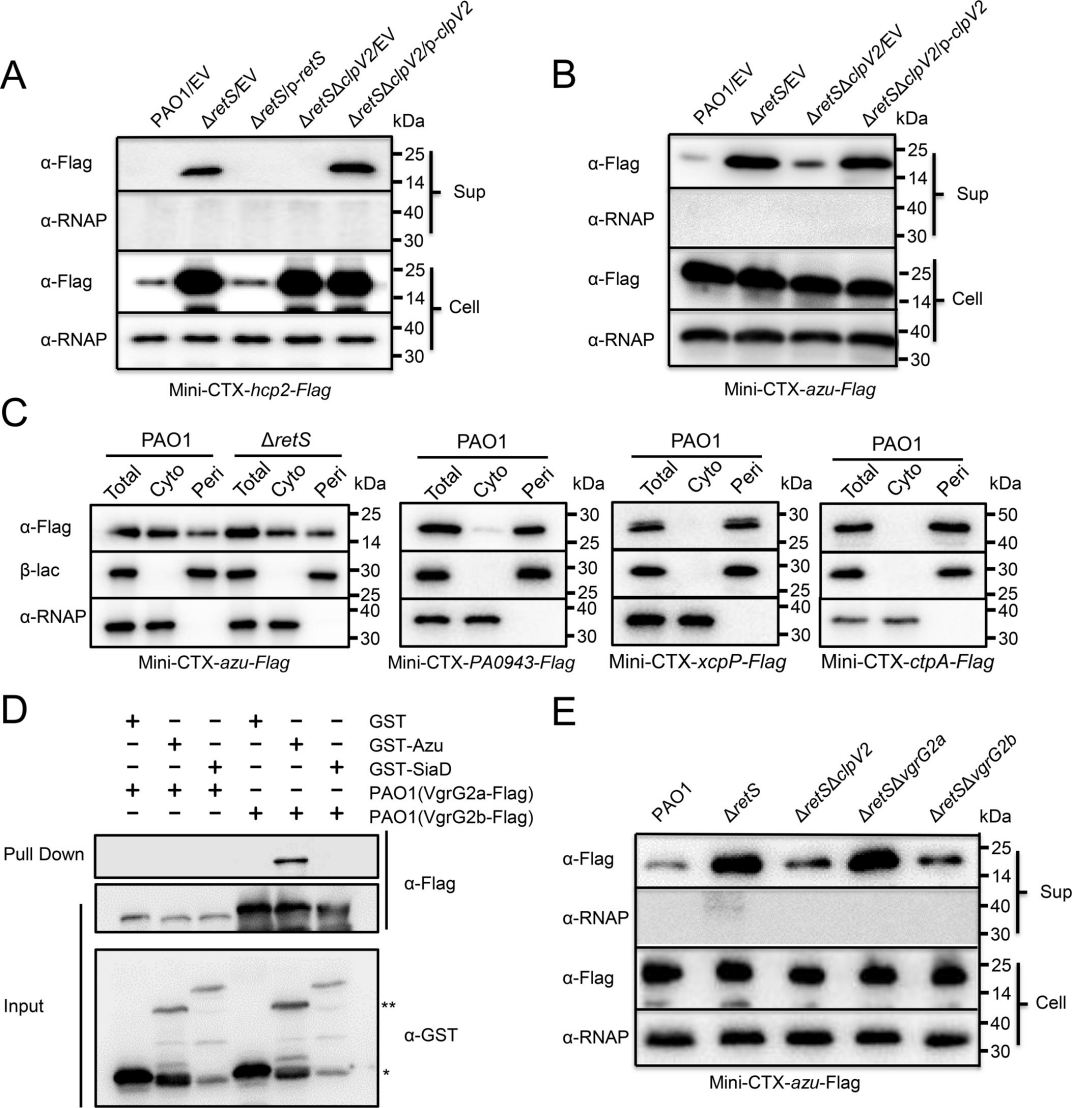
H2-T6SS is required for Azu secretion. **(A)** Deletion of *retS* enables expression and secretion of Hcp2. A mini-CTX plasmid directing the expression of Hcp2-Flag chimera was integrated into the *P*. *aeruginosa* derivative strains, respectively. Western blot analysis of Hcp2-Flag in the cell-associated (Cell) and concentrated supernatant (Sup) protein fractions from the indicated strains grown in M9 minimal medium. For the pellet fraction, an antibody against RNA polymerase α (α-RNAP) was used as a loading control in this and subsequent blots. **(B)** Azu is secreted by H2-T6SS. The cultured condition is the same as described in A. EV represents the empty vector pAK1900. **(C)** Total cell (Total), Cytoplasmic (Cyto) and periplasmic (Peri) fractions of *P*. *aeruginosa* expressing Azu, PA0943, XcpP or CtpA were examined by Western blot. The tagged proteins were detected using a Flag antibody. RNA polymerase (RNAP) and β-lactamase (β-lac) were used as cytoplasmic or periplasmic fraction controls, respectively. Data are representative of three independent replicates. **(D)** Interactions of Azu and VgrG2b. Cell lysates of *P*. *aeruginosa* containing pMMB67H-VgrG2a-Flag or pMMB67H-VgrG2b-Flag were incubated with GST or GST-Azu protein individually, and protein complex were captured by glutathione beads. The single and double asterisks represent GST and GST-Azu, respectively. GST-SiaD was a negative control. **(E)** Azu secretion is VgrG2b dependent but not VgrG2a. The cultured condition is the same as described in A.

Expression of the T6SS is regulated by multiple proteins at the transcriptional and posttranscriptional levels [25–28]. In contrast to the wild-type parent, the strain lacking RetS sensor, which regulates the two-component GacS/GacA and Rsm signaling pathway, has higher expressions of H1- and H2-T6SS [25, 26, 29]. To further probe whether the H2-T6SS is regulated by RetS under different growth conditions, the secretion of Hcp2 was determined in Δ*retS* and Δ*retS*Δ*clpV2* strains cultured in M9 minimal medium. Consistent with observations under lysogeny broth (LB) conditions [25, 26] (S1A Fig), deletion of *retS* strongly promoted Hcp2 secretion relative to the parental strain in M9 minimal medium (Fig 1A). After complementation with a plasmid carrying a wild-type *retS* gene (p-*retS*), Hcp2 production was inhibited to wild-type levels in the Δ*retS* mutant. The Δ*retS*Δ*clpV2* double mutant abrogated Hcp2 secretion, and this effect was fully complemented by ectopic expression of *clpV2* (Fig 1A and S1A Fig). PldA, identified as an effector for the H2-T6SS, is encoded remotely from the H2-T6SS cluster, within the orphan VgrG4b cluster [30]. To further explore the function of RetS on H2-T6SS effector secretion, we engineered a PldA-Flag fusion and monitored its production by western blot analysis in M9 minimal medium. PldA secretion is regulated by RetS (S1B Fig). Likewise, secretion of VgrG2b is also dependent on RetS (S1C Fig). These results confirm that the RetS sensor controls the expression and activity of H2-T6SS. Deletion of *clpV2* should disable the T6SS as well as prevent re-firing and secretion of these proteins in a PAO1 Δ*retS* strain. Using these backgrounds, the following mass spectrometric secretome analysis was performed.

### Mass spectrometric analysis of H2-T6SS-dependent secretomes

Next, we applied an MS-based method of secretome analysis to identify uniquely secreted substrates between the Δ*retS* and Δ*retS*Δ*clpV2* strains. To avoid the effects of environmental factors, we collected secretome samples from cells cultured in M9 minimal medium. The proteins present in such samples from the Δ*retS* and Δ*retS*Δ*clpV2* strains were then analyzed by liquid chromatography-tandem mass spectrometry (LC-MS/MS). The distribution of ratios (Δ*retS*Δ*clpV2/*Δ*retS*) of these proteins was measured to determine whether each protein was differentially secreted by the H2-T6SS. Since we did not predict the H2-T6SS to exhibit a global effect on the secretome, less abundant proteins (ratio< 0.5) were selected. The results of the MS analyses are summarized in S1 Table. A total of 21 proteins were identified that met our filtering criteria. As expected, most of the known H2-T6SS-secreted substrates, such as Hcp, VgrG2a, VgrG2b, Tle3 and Tle4, were present among our data (S1 Table). The most abundant H2-T6SS-dependent substrates were categorized as transporters and hypothetical proteins with uncharacterized functions. Proteins whose abundances were much lower in the Δ*retS*Δ*clpV2* mutant than in the Δ*retS* background (ratio< 0.5) were considered as potential H2-T6SS effector candidates in *P*. *aeruginosa*. Altogether, this is the first identification of the H2-T6SS-dependent secretome, which will assist us in future investigations of the diverse roles of the H2-T6SS.

### Cu-binding protein Azu is secreted by H2-T6SS

Azu is a Cu-binding protein found on a locus in an unlinked site that lacks other apparent T6SS elements [10, 30]. The abundance of Azu was much higher in the Δ*retS* mutant than in the Δ*retS*Δ*clpV2* strain (S1 Table). To confirm the MS results, the *azu* gene (PA4922) was fused with a Flag-tag sequence at the C-terminus in the Mini-CTX-lacZ plasmid, and the fusion (*azu*-Flag) was integrated into the wild-type PAO1, Δ*retS*, and Δ*retS*Δ*clpV2* chromosomes, respectively. Next, we compared the locations of Azu in these strains. Consistent with our MS findings, western blot analysis of cell and supernatant fractions with anti-Flag antibody showed that secretion of the Azu protein was strongly improved in the Δ*retS* mutant compared to that in the wild-type PAO1 (Fig 1B). However, deletion of *clpV2* in the Δ*retS* background strain significantly reduced the Azu secretion (Fig 1B). Bioinformatic analysis revealed that Azu harbors a signal peptide and has been shown to be a periplasmic protein [31]. Generally, the Sec system recognizes the signal peptide during translation of the nascent peptide and transports the unfolded protein into the periplasm [32]. However, the azurin protein with the signal peptide is secreted by T6SS. We postulate that the azurin protein might be also localized in the cytoplasm. To verify this hypothesis, we performed sub-cellular fractionation experiments. Interestingly, our data showed that the distribution of azurin was observed both in the periplasmic and cytoplasmic compartments, which is different from the previous reports that proteins with the Sec signal peptide should be located in the periplasm (Fig 1C) [33–35]. To confirm the effect of the signal peptide on its secretion, we tested the Azu_ss_-Flag (Azu lacking the signal peptide) levels in the supernatant of the wild-type PAO1, Δ*retS*, and Δ*retS*Δ*clpV2* strains. Like to the Azu, the secretion of the Azu_ss_-Flag was dependent on H2-T6SS (S2 Fig), which is consistent with previous report that T6SS is thought to be as a Sec-independent secretion system [36].

ClpV is predicted to function as an energizing component [18]. We then tested the ability of the ClpV2 E286A/E692A variant deficient in ATP hydrolysis, which harbors mutations in the Walker B motifs of both AAA domains. Complementation with the p-*clpV2m* (E286A/E692A) did not restore Azu secretion (S3A Fig). We also observed that strains lacking *hcp2* displayed markedly reduced Azu secretion (S3B Fig). In addition, Azu secretion was impaired in the deletion mutant of *tssM2*, encoding a key T6SS structural protein [18], and restored in the complemented strain (S3B Fig). However, the secreted level of Azu was not affected by ClpV1 or ClpV3 (S3C Fig), demonstrating that the H1-T6SS and H3-T6SS are not required for Azu secretion, as it is H2-T6SS-specific.

To identify the secretion route of Azu, we examined the interactions between Azu and VgrG, the T6SS component that acts as the carrier for effector secretion by direct binding [37]. Glutathione S-transferase (GST)-Azu was able to retain VgrG2b but GST was not. In contrast, no interaction was observed between GST-Azu protein and VgrG2a (Fig 1D). In addition, Azu secretion was VgrG2b dependent but not VgrG2a dependent (Fig 1E). Thus, Azu is likely a substrate of the H2-T6SS; and its secretion is VgrG2b-dependent.

The relationship between Azu and ClpV2 led us to test whether Azu and ClpV2 are required for Cu^2+^ acquisition. Therefore, we measured the total metal content in bacterial cells using inductively coupled plasmon resonance atomic absorption mass spectrometry (ICP-MS). Our results showed that deletion of the *azu* or *clpV2* genes significantly lowered intracellular Cu^2+^ levels (S3D Fig), indicating that Azu and ClpV2 are involved in Cu^2+^ uptake.

### H2-T6SS is activated and confers a competitive advantage under low-Cu^2+^ conditions

**Fig 2:**
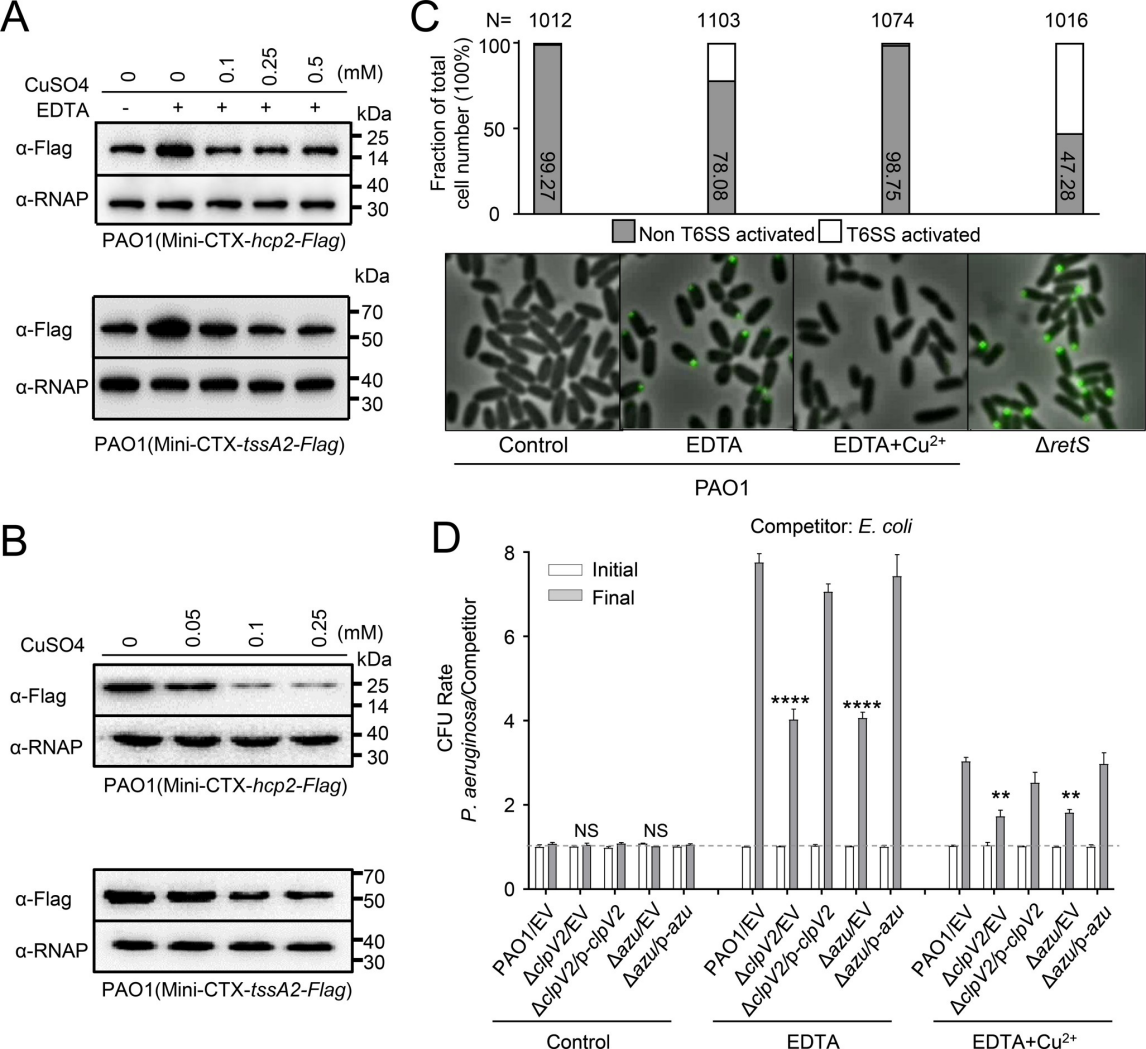
Copper (Cu^2+^) regulates H2-T6SS expression and promotes a growth advantage of *P*. *aeruginosa* for bacterial competition. **(A-B)** The expression of *hcp2* and *tssA2* was induced by low Cu^2+^. A mini-CTX plasmid directing the expression of Hcp2-Flag or TssA2-Flag chimera was integrated into the *P*. *aeruginosa* background strain, respectively. **(A)** Bacteria was cultured in LB medium supplemented with either 0.25 mM EDTA or 0.25 mM EDTA with the indicated concentrations of CuSO_4_. Total protein was probed for the presence of the fusion protein. **(B)** Bacteria was cultured in LB medium supplemented with either 0.05 mM, 0.1 mM, or 0.25 mM CuSO_4_. Total protein was probed for the presence of the fusion protein. **(C)** Cu^2+^ influences H2-T6SS assembly. Chromosomally encoded ClpV2-sfGFP localization in the *P*. *aeruginosa* measured by fluorescence microscopy. Cells were grown in the indicated conditions to OD_600_ = 1.0 and H2-T6SS activated were analyzed. N = total number of cells analyzed for each strain. (**D)** Interbacterial growth competition assays between *P*. *aeruginosa* and *E*. *coli*. The competition assays were examined under LB conditions supplemented with either 0.25 mM EDTA or 0.25 mM EDTA and 0.1 mM CuSO_4_ for 6 h. Quantification of cfu before (initial) and after (final) growth competition assays between the indicated organisms. Note that wild-type PAO1 displayed a growth advantage against the competitors than the Δ*clpV2* and Δ*azu* mutant under the present conditions. Error bars represent the mean ± s.d. of three independent experiments. **P<0.01, ****P<0.0001 based on two-way ANOVA Dunnett’s multiple comparison test; NS, not significance. EV represents the empty vector pAK1900.

We next attempted to evaluate whether the H2-T6SS is influenced by Cu^2+^. To this end, the protein levels of Hcp2 and TssA2 were determined by western blot analysis. Supplementing the growth medium with 0.25 mM EDTA strongly enhanced Hcp2 and TssA2 production, and this activity was repressed by Cu^2+^ (Fig 2A). By contrast, production was not repressed by zinc or calcium ions (S4A Fig). Expression of Hcp2 and TssA2 was repressed by Cu^2+^ in a concentration-dependent manner (Fig 2B). Consistently, the Azu was more secreted under copper starvation than in rich Cu^2+^ medium (S4B Fig). However, no differences were observed with Hcp1 or Hcp3 activity under the same conditions (S4C Fig). Altogether, these results demonstrate that Cu^2+^ regulates H2-T6SS and Azu secretion.

ClpV2 is an essential component of the secretion apparatus [18], and the effect of Cu on the H2-T6SS should be reflected in the subcellular localization of ClpV2. To visualize ClpV2, we engineered a C-terminal fusion of the green fluorescent protein (sfGFP) to ClpV2 (ClpV2-sfGFP) and integrated it into the wild-type PAO1 and the *retS* mutant, respectively. As expected, ClpV2-sfGFP localized to single discrete foci in the majority of the Δ*retS* mutant cells (Fig 2C). Importantly, the number of cells with discrete ClpV2-sfGFP foci was drastically increased when the bacteria were cultured in LB medium supplemented with 0.25 mM EDTA. Instead, the punctate localization pattern of ClpV2-sfGFP was similar to that of the wild type after addition of 0.1 mM Cu^2+^ in the above conditions (Fig 2C; S1 Movie), but not by zinc or calcium ions (S5A Fig; S2 Movie). Neither EDTA nor Cu^2+^ affected the stability of ClpV2-sfGFP (S5B Fig). Thus, this result suggests that Cu appears to be required for punctate ClpV2-sfGFP localization in the wild-type background.

Cu not only regulates H2-T6SS activity but also affects its assembly, suggesting that it may play a role in bacterial competition for essential nutrients. To test this hypothesis, we performed interbacterial growth competition assays between *P*. *aeruginosa* derivative strains and *Escherichia coli*. Our results revealed that all *P*. *aeruginosa* derivative strains displayed a growth advantage in LB medium containing 0.25 mM EDTA, but the competitive advantage of the wild-type parent over *E*. *coli* DH5α was largely decreased in the Δ*clpV2* and Δ*azu* strains (Fig 2D). In addition, this growth advantage was inhibited by the addition of 0.1 mM Cu^2+^. These data indicate that the H2-T6SS/Azu-mediated Cu^2+^ uptake confer a competitive advantage.

### CueR directly regulates H2-T6SS activity

CueR is a MerR-type transcriptional regulator involved in Cu homeostasis in *P*. *aeruginosa* [12]. Therefore, we hypothesized that the expression of *azu* may be regulated by CueR. To address this possibility, we engineered a *cueR* deletion mutant and a *cueR*-overexpressing plasmid (p-*cueR*). Loss of *cueR* resulted in increased levels of *azu* transcription and protein production, and complementation with a p*-cueR* plasmid restored *azu* activity to wild-type levels (S6A and S6B Fig).

**Fig 3:**
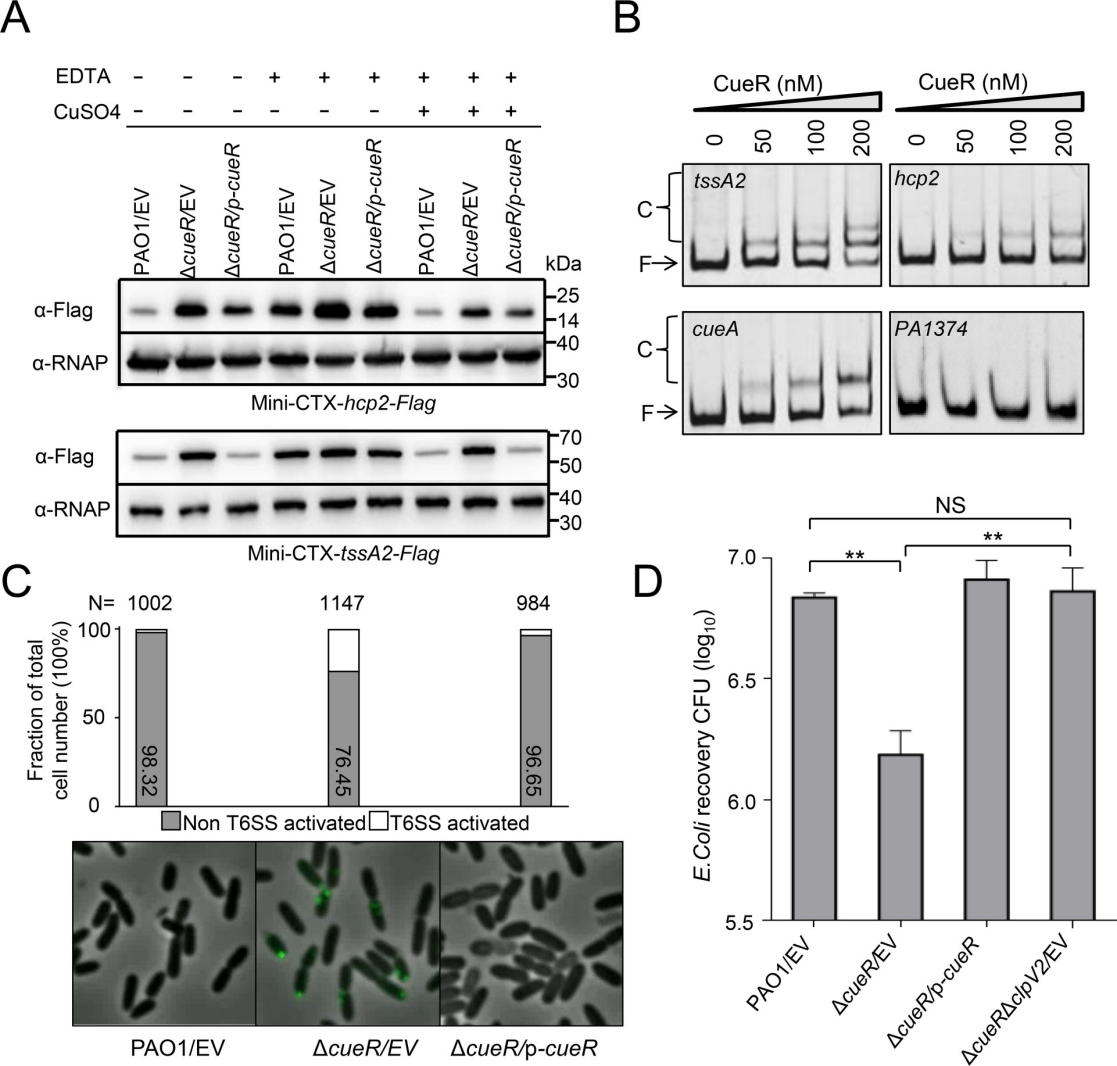
CueR regulates the expression of H2-T6SS directly and is required for H2-T6SS-mediated antibacterial activity. **(A)** Deletion of *cueR* increases the levels Hcp2 (upper) and TssA2 (down) relative to wild-type PAO1. Western blot analysis of Hcp2-Flag and TssA2-Flag in the cell-associated fractions from wild-type PAO1, Δ*cueR*, and the complemented strain (Δ*cueR/*p*-cueR*) cultured in LB medium containing 0.25 mM EDTA with or without 0.1 mM CuSO_4_. **(B)** EMSAs showing that CueR binds to the promoter region of *hcp2* and *tssA2*. PCR products containing *hcp2*, *tssA2*, *cueA*, and *PA1374* promoter fragments were added to the reaction mixture at a concentration of 2.0 ng. The protein concentration of each sample is indicated above its lane. As a positive control, CueR can efficiently bind to the *cueA* promoter region. However, no band shift was observed when CueR protein incubates with PA1374 promoter region. **(C)** CueR affects H2-T6SS assembly. Chromosomally encoded ClpV2-sfGFP localization in the wild-type PAO1 and Δ*cueR* mutant measured by fluorescence microscopy, respectively. Cells were grown in LB to OD_600_ = 1.0 and H2-T6SS activated were analyzed. N = total number of cells analyzed for each strain. **(D)** Quantification of recovered prey cells (*E*. *coli*) after co-incubation with strains PAO1, Δ*cueR*, or Δ*cueR*Δ*clpV2*. Error bars indicate the mean ± s.d. of three biological replicates. **p<0.01 by student′s *t*-test; NS, not significant when compared to prey recovery after co-incubation with the parent strain. EV represents the empty vector pAK1900.

Given that activity of the H2-T6SS was induced by low Cu^2+^, we then determined the effect of CueR on H2-T6SS expression. Western blot analysis using anti-Flag antibody showed that deletion of *cueR* increased Hcp2 and TssA2 protein levels relative to the parental strain (Fig 3A and S6C Fig). The activity of these genes in the Δ*cueR* mutant was almost completely alleviated by complementation of the *cueR* gene (Fig 3A and S7A Fig), further supporting the role of CueR in H2-T6SS expression. Additionally, the expression of *hcp2* and *tssA2* in the Δ*cueR* mutant was slightly response to the same concentrations of Cu^2+^, indicating that this regulation by Cu^2+^ is mainly dependent on CueR (Fig 3A and S7A Fig). However, no significant differences in Hcp1 or Hcp3 levels were observed when comparing the wild-type PAO1 and *cueR* mutant (S7B Fig). Therefore, we conclude that CueR only impacts H2-T6SS activity, but not the H1- and H3-T6SSs.

Since CueR is a negative regulator for the H2-T6SS, we next assessed whether this was a direct effect using electrophoresis mobility shift assays. A GST-tagged CueR protein was purified and the GST tag was removed by incubation with PreScission Protease. DNA fragments known to bind or not bind with CueR were selected as controls [38]. Similar to the positive control of *cueA*, which is involved in copper export and directly regulated by CueR [39], incubation of probes containing *hcp2* (−485 to -1 relative to the ATG start codon) or *tssA2* (−659 to -1 relative to the ATG start codon of the first open reading frame of the H2-T6SS operon) promoter sequences with CueR led to the formation of protein-DNA complexes (Fig 3B). To further identify the CueR binding motif, we generated a series of truncated intergenic region fragments of *hcp2* or *tssA2* and used them to perform EMSAs. We showed that CueR bound to the sequence of *tssA2* (AATAGGAAAATTCCCAAAGAGGGAATGTCCTGTT, -173 to -140 relative to the ATG start codon) or *hcp2* (TCCGAGAGAGTGCGCAACTTTTTGCAAGCGGTGCGCAAAAAGTTGCGCAA, -208 to -159 relative to the ATG start codon) (S7C Fig). However, these two sequences are different from the known CueR-binding sites [38], indicating its diverse functions. Overall, these results suggest that CueR regulates H2-T6SS expression directly.

### CueR is required for H2-T6SS-mediated antibacterial activity

We next questioned whether CueR might be required for the localization of ClpV2-sfGFP. To address this, ClpV2-sfGFP was integrated into the Δ*cueR* mutant background. Importantly, the frequency of punctate foci was more in the Δ*cueR* mutant relative to those in wild-type parent (Fig 3C; S3 Movie). This result indicates that deletion of *cueR* activates the expression of the apparatus, thus promoting assembly.

Since mutation of the *cueR* gene influences H2-T6SS activity and assembly, we then determined whether CueR was required for H2-T6SS-mediated antibacterial activity. Therefore, we co-incubated the Δ*cueR* mutant killer strain with prey cells (*E*. *coli*). Our results showed that the Δ*cueR* mutant significantly increased the killing of prey cells relative to the parent control, whereas the Δ*cueR*Δ*clpV2* mutant abrogated the killing activity (Fig 3D), suggesting that CueR mediated H2-T6SS-dependent antibacterial activity.

### Azu interacts with a Cu^2+^-related OprC

**Fig 4:**
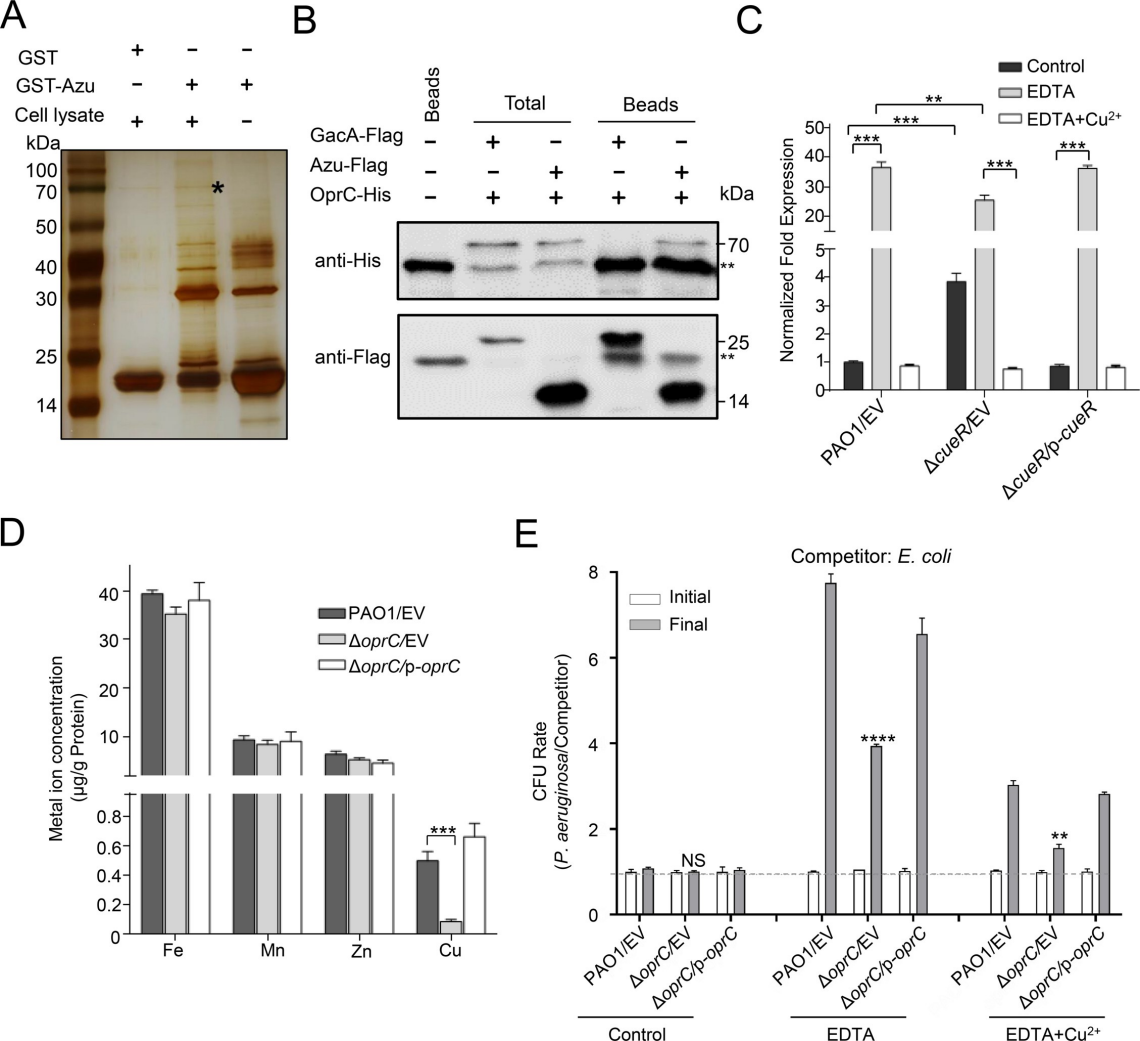
Azu interacts with a TBDT family transporter involved in Cu^2+^ transport. **(A)** OprC was retained by agarose beads coated with GST-Azu. GST-binding beads coated with GST-Azu or GST was incubated with cell lysates. After washing with PBS buffer, the proteins separated by SDS/PAGE gels were visualized using silver staining, and bands retained by the GST-Azu coated beads were identified by mass spectrometry. The asterisk band represents the OprC protein. **(B)** Co-IP assays showing Azu binds to OprC. Cell lysates of *P*. *aeruginosa* containing pMMB67H-*oprC*-His with either p-*gacA*-Flag or p-*azu*-Flag were individually incubated with Flag beads, and then the beads retained protein complexes were detected by western blot against Flag antibody or His antibody. The double asterisks denote a nonspecific signal in the soluble protein fraction. Beads only sample was a blank control, GacA-Flag was a negative control. **(C)** The expression of *oprC* was repressed by CueR and high Cu^2+^. Cells of the indicated strains were grown in LB medium containing 0.25 mM EDTA with or without 0.1 mM CuSO_4_. The expression of *oprC* was measured by qRT-PCR. **(D)** OprC is involved in Cu^2+^ acquisition in *P*. *aeruginosa*. Strains were cultured at OD_600_ = 1.0 in M9 medium containing 1.0 mM EDTA. Cu^2+^ associated with bacterial cells was measured by ICP-MS. **(E)** Interbacterial growth competition assays between *P*. *aeruginosa* and *E*. *coli*. The competition assays were examined in LB or LB medium containing 0.25 mM EDTA with or without 0.1 mM CuSO_4_ for 6 h. Quantification of cfu before (initial) and after (final) growth competition assays between the indicated organisms. **(C-E)**, error bars represent the mean ± s.d. of three biological replicates, **P<0.01, ***P<0.001, ****P<0.0001 based on two-way ANOVA Dunnett’s multiple comparison test. NS indicates not significance. EV represents the empty vector pAK1900.

To further reveal how Azu transports Cu^2+^ into the cell, we attempted to identify bacterial components that interact with Azu. We tested this using the protein pull-down assay by incubating GST-Azu or GST with cell lysates of *P*. *aeruginosa* strains. The mixtures were further purified by GST beads and proteins retained on the beads were separated by sodium dodecyl sulfate polyacrylamide gel electrophoresis (SDS-PAGE) and then visualized by silver staining. A protein band was retained by beads coated with GST-Azu, but not by GST alone (Fig 4A). MS analysis showed that the most abundant protein encodes a copper transport outer membrane porin (OprC, PA3790) (S2 Table). To verify the interaction between Azu and OprC, we performed the Co-IP assays *in vivo*. Western blot result showed that a specific interaction between Azu and OprC was observed (Fig 4B and S8A Fig). The interaction was further confirmed by the binding assay with purified GST-Azu and OprC-His_6_ proteins *in vitro* (S8B Fig).

Given that OprC interacts with Azu, we then analyzed the effect of Cu^2+^ on *oprC* expression. As shown in Fig 4C, the activity of *oprC* was induced by low Cu^2+^. Indeed, CueR could bind to the *oprC* upstream region (S8C Fig), thus directly repressing *oprC* expression and its protein levels (Fig 4C and S8D Fig). Sequence analysis of OprC predicted a 107-residue N-terminal TonB-plug domain (residues 75–181) and a TonB-dependent receptor domain (residues 224–722) (S9A Fig), indicating that OprC may be involved in transportation of Cu^2+^. To evaluate the role of OprC in Cu^2+^ transport, the total metal content of bacterial cells was measured by ICP-MS. Deletion of *oprC* drastically reduced intracellular Cu^2+^ levels compared to those of wild-type PAO1, and complementation of the gene restored wild-type levels (Fig 4D). However, mutation of the *oprC* gene had little effect on the accumulation of iron, manganese, and zinc ions.

We next performed interbacterial growth competition assays to confirm the importance of OprC in mediating the Cu^2+^ transport activity of the H2-T6SS. Phylogenetic analysis showed that OprC homologs were widely distributed in *Pseudomonas* species, but absent in *E*. *coli* DH5α (S9B Fig). As shown in Fig 4E, the *P*. *aeruginosa* wild type was highly competitive against the *E*. *coli* DH5α competitor in LB medium supplemented with 0.25 mM EDTA. However, the competitive advantage of the *P*. *aeruginosa* wild type over *E*. *coli* DH5α was largely decreased in the Δ*oprC* mutant. In addition, this growth advantage was decreased by supplementation with 0.1 mM Cu^2+^. Together, these data indicate the critical role of OprC in H2-T6SS-dependent Cu^2+^ acquisition during bacterial competition.

### The H2-T6SS-dependent transport system is critical for *P*. *aeruginosa* in bacterial competition and virulence

**Fig 5:**
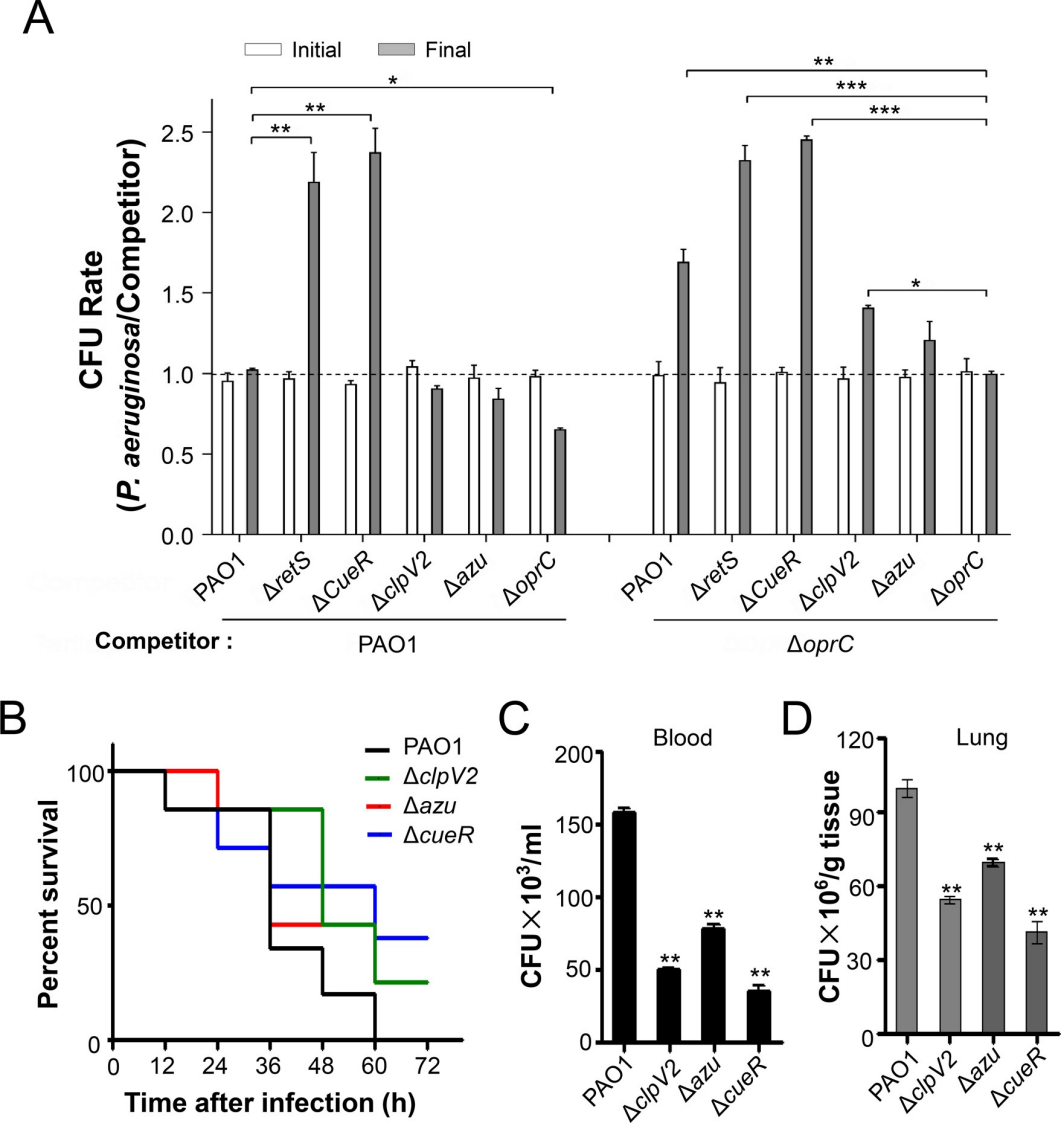
H2-T6SS-dependent Cu^2+^ transport system is important for competitive growth and virulence. **(A)** OprC and Azu mediate H2-T6SS-dependent intrabacterial competition. The indicated strains were grown in LB medium supplemented with 0.25 mM EDTA for 6 h. Quantification of cfu before (initial) and after (final) growth competition assays between the indicated organisms. The cfu of the relevant *P*. *aeruginosa* versus competitor was plotted. Error bars represent the mean ± s.d. of three biological replicates. *P<0.05, **P<0.01, ***P<0.001 based on two-way ANOVA Dunnett’s multiple comparison test. **(B)** The *clpV2*, *azu*, and *cueR* deletion reduced the virulence of *P*. *aeruginosa*. C57BL6 mice were intranasally challenged with wild-type PAO1, Δ*clpV2*, Δ*azu*, or Δ*cueR* at 1×10^7^ cfu in 50 μl PBS, moribund mice were killed to obtain survival data (n = 6/strain). **(C-D)** Mice were infected with 1×10^7^ wild-type PAO1, Δ*clpV2*, Δ*azu*, or Δ*cueR* strain intranasally (n = 6/strain). At 12 h, serums and lungs from mice infected with no bacteria (PBS), or the indicated strains were recovered. Bacterial loads were determined by serial dilution and plating. **P<0.01 compared to wild-type PAO1 by Student's *t* test. The strains contain either the empty vector pAK1900 or the complemented plasmid.

To further determine whether OprC mediates the Cu^2+^ transport activity of Azu and the H2-T6SS, we performed intrabacterial growth competition assays between *P*. *aeruginosa* and wild type PAO1 or Δ*oprC* mutant under Cu^2+^-limited conditions. Compared to wild-type parent, the competitive growth advantage was decreased in the cells lacking OprC or Azu due to they did not update Cu^2+^. However, the competitive advantages were increased in the Δ*cueR* and Δ*retS* mutants under Cu^2+^-limited conditions (Fig 5A). This result indicates that OprC mediated Cu^2+^ transport by binding to Azu, which secreted by itself or other bacteria.

Cu^2+^ transport systems have been shown to be required for full virulence of multiple bacterial pathogens [40, 41]. To investigate the role of H2-T6SS-dependent transport systems in pathogenesis, we determined the virulence of *P*. *aeruginosa* strains in a mouse model of acute pneumonia. The acute infection model has been used previously to assess the virulence of T6SSs in *P*. *aeruginosa* [27, 42]. Therefore, 6-week-old C57BL/6 mice were intranasally inoculated with 1×10^7^ cells of wild-type PAO1, Δ*clpV2*, Δ*azu*, or Δ*cueR* mutants respectively. Survival analysis showed that loss of *clpV2*, *azu*, or *cueR* significantly increased mouse survival compared to the wild-type PAO1. The Δ*clpV2*, Δ*azu*, and Δ*cueR* strains caused 20%, 67%, and 50% of mice to die by 36 h, respectively, and 25% of mice infected with these three mutants were alive at 60 h. Meanwhile, 67% of mice infected with wild-type PAO1 were dead by 36 h, and 100% were dead by 60 h (Fig 5B). We also determined bacterial loads in the blood and lungs of mice infected with the above *P*. *aeruginosa* strains (Fig 5C and 5D). In summary, these results indicate that the H2-T6SS-dependent Cu^2+^ transport system is important for bacterial virulence.

## Discussion

**Fig 6:**
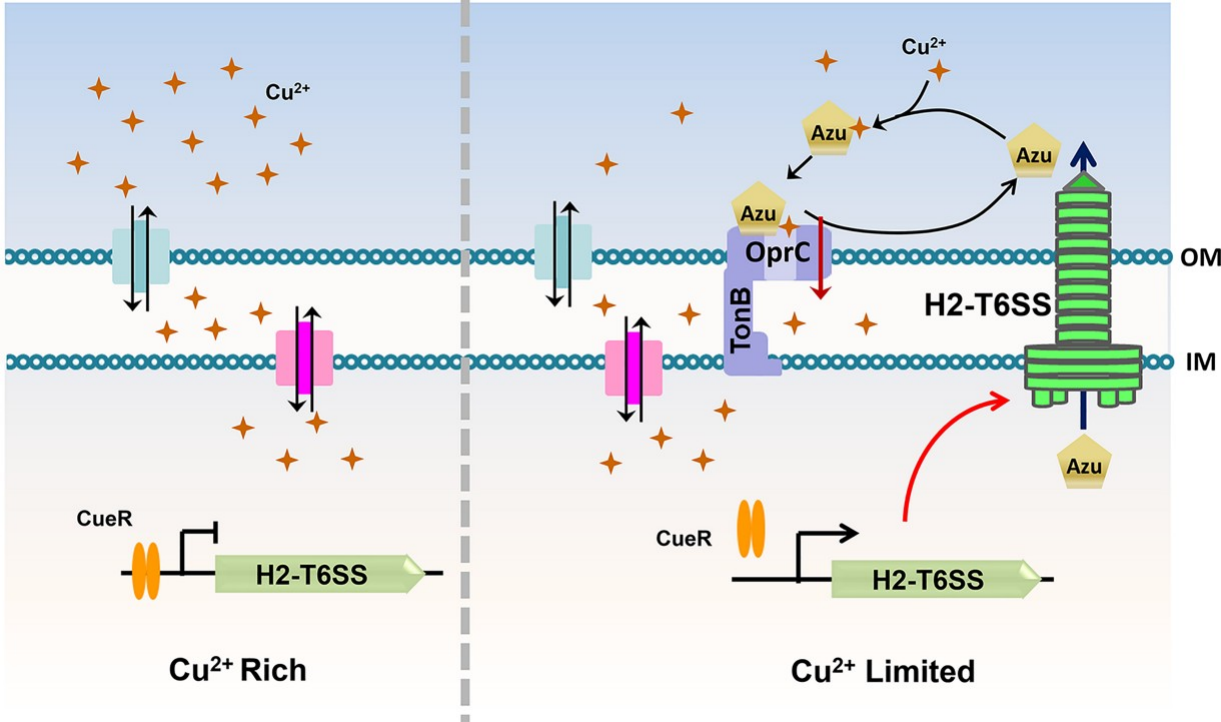
A proposed model of H2-T6SS-mediated Cu^2+^ transport in *P*. *aeruginosa*. Under Cu^2+^ rich conditions, the copper homeostasis regulator CueR represses the expression of H2-T6SS genes. The Cu^2+^ transport is mainly dependent on the known systems, such as CopY, CueA and CusR/S. However, the repression of H2-T6SS by CueR is relieved under the Cu^2+^-limited conditions, and the Cu^2+^-binding protein Azu is subsequently secreted by the activated H2-T6SS. Secreted Azu scavenges extracellular Cu^2+^ and delivers its load via direct interaction with OprC.

The T6SS is widely conserved among Gram-negative bacteria and is a central determinant of bacterial fitness in polymicrobial communities. In recent years, progress on the T6SS has been made in determining its structure and biogenesis in *P*. *aeruginosa*, but the function of the T6SS remains largely unknown. Similar to any secretion system, identification and characterization of the protein substrates that it exports are key to understanding its functions. Particularly, the identities of genuine substrates of the H2- and H3-T6SSs are poorly documented due to the lack of available conditions for their expression. In accordance with recent reports [25, 26], we demonstrated here that deletion of the *retS* sensor fully activates H2-T6SS secretion in *P*. *aeruginosa* in M9 minimal medium (Fig 1A and S1B and S1C Fig). Accordingly, we performed proteomic analyses and identified 21 H2-T6SS-dependent secretion substrates (S1 Table). We further characterized one substrate of the H2-T6SS, the Cu-binding protein Azu, and proposed a model for Cu^2+^ transport through the outer membrane mediated by OprC and the effector Azu. When *P*. *aeruginosa* is grown under low-Cu^2+^ conditions, the H2-T6SS is induced expression resulting in enhanced activity to secrete Azu into the extracellular milieu. Secreted Azu acquires extracellular Cu^2+^ and delivers its load via direct interaction with OprC (Fig 6). The T6SS-Azu-OprC system represents a new system for Cu homeostasis in cells. This T6SS-mediated Cu^2+^ uptake model expands our understanding of the diverse functions of T6SSs.

Previous study shows that Azu harbors a peptide signal and is located in periplasm [31]. In this study we found that Azu is distributed in the periplasmic and cytoplasmic compartments both, which is different from the previous reports that proteins with the sec signal peptide should be located in the periplasm (Fig 1C) [32–35]. Therefore, the azurin protein in the cytoplasm interacts with VgrG2b and then is secreted by T6SS (Fig 1D and 1E). Why and how the Azu protein located in the cytoplasm needs to be further investigated. It is noted that a *Vibrio parahaemolyticus* toxin is exported by the Sec and type III secretion machineries in tandem [43], suggesting a potential interaction among these secretion systems.

Bacteria have evolved many effective systems to cope with nutritional challenges, such as scavenging metal ions from the environment. Similar to TseM, a T6SS substrate involved in manganese uptake in *Burkholderia thailandensis* [44], the Azu protein appears to be a substrate of the H2-T6SS that functions by interacting with the TBDT OprC. Here, we provide evidence that Azu facilitates OprC-mediated Cu^2+^ transport. First, the *azu* mutant was deficient in Cu^2+^ accumulation (S3D Fig); second, the secretion of *azu* was strongly induced by low Cu^2+^ concentrations (S4B Fig); third, Azu directly interacts with OprC (Fig 4A and 4B); and finally, the function of Azu in bacterial competition is dependent on OprC (Fig 5A). In addition, the role of the H2-T6SS and its substrate Azu in Cu acquisition is further supported by the fact that the activity of H2-T6SS-related genes is activated by low Cu^2+^ and repressed by high Cu^2+^ (Figs 2A and 3A). Altogether, we conclude that the H2-T6SS-secreted Cu^2+^-binding protein, Azu, sequesters Cu^2+^ from the environment and interacts with the outer membrane transporter, OprC, for improved Cu^2+^ uptake.

It has recently been reported that the T6SS is involved in zinc ion uptake in *Yersinia pseudotuberculosis* [45] and mediates accumulation of manganese ions under oxidative stress in *B*. *thailandensis* [44]. In *P*. *aeruginosa*, the T6SS effector TseF recruits Pqs-containing outer membrane vesicles for iron acquisition [24]. Similarly, the T6SS in *Pseudomonas taiwanensis* is also involved in secretion of the iron chelator pyoverdine by an unknown mechanism [46]. In addition, the expression of several T6SS-related genes is regulated by iron and zinc in *Burkholderia mallei* and *Burkholderia pseudomallei* [47], and by the ferric uptake regulator Fur in *P*. *aeruginosa* [27]. Our finding of the involvement of the T6SS in Cu^2+^ uptake further supports the role of the T6SS in metal ion acquisition and expands the range of known functions of this secretion system. Therefore, T6SS-mediated metal transport may represent a novel mechanism by which the T6SS secrets metal-scavenging proteins into the extracellular medium via interaction with specific outer membrane transporters to maintain metal ion homeostasis in cells. These data exemplify how *P*. *aeruginosa* deploys the T6SS to adapt to complex environments.

In conclusion, we revealed a relatively comprehensive secretome of the H2-T6SS by proteomic analysis and demonstrated how the *P*. *aeruginosa* T6SS deploys Cu to take a growth advantage in bacterial competition. Future studies will focus on functional characterization of other H2-T6SS effectors to expand our understanding of the diverse roles of the T6SS in bacteria.

## Materials and methods

### Ethical statement

All animal experiments were performed in accordance with the US National Institutions of Health animal care and institutional guidelines. Animal handling protocols were approved by the University of North Dakota Institutional Animal Care and Use Committee (IACUC). The UND Animal Facilities were accredited by the Association for Assessment and Accreditation of Laboratory Animal Care International (AAALAC Assurance Number: A3917-01).

### Bacterial strains and growth conditions

The bacterial strains and plasmids used in this study are listed in S3 Table. *P*. *aeruginosa* PAO1 and derivatives were grown in LB broth or M9 minimal medium with shaking at 220 rpm at 37°C. For proteomic analysis, strains were cultured in M9 minimal medium. For plasmid maintenance, antibiotics were used at the following concentrations where appropriate: for *E*. *coli*, gentamicin at 15 μg/mL, ampicillin at 100 μg/mL, and tetracycline at 10 μg/mL; for *P*. *aeruginosa*, gentamicin at 50 μg/mL in LB or 150 μg/mL in *Pseudomonas* isolate agar (PIA), tetracycline at 150 μg/mL in LB or 300 μg/mL in PIA, and carbenicillin at 500 μg/mL in LB.

### Construction of plasmids

Plasmid p-*clpV2* was constructed by PCR amplification of the *clpV2* gene using the primers p-*clpV2*-S/p-*clpV2*-overlap-A and p-*clpV2*-overlap-S/p-*clpV2*-A (S4 Table). The PCR products were digested with the indicated enzymes and cloned into pAK1900 [48]. Plasmids p-*hcp2*, p-*tssM2*, p-*cueR*, and p-*azu* were constructed using similar methods.

Plasmid pUCP26-*clpV2* was constructed by PCR amplification of the *clpV2* gene using the primers p-*clpV2*-S/p-*clpV2*-overlap-A and p-*clpV2*-overlap-S/p-*clpV2*-A (S4 Table). The PCR products were digested with the indicated enzymes and cloned into pUCp26.

The Mini-*hcp2*-Flag-S/A primer pair was used to amplify the DNA fragment encoding *hcp2* gene driven by its promoter that intended to fuse with a C-terminal Flag-tag by PCR. The digested products were cloned into the Mini-CTX-*lacZ* plasmid to generate Mini-CTX-*hcp2*-Flag. These plasmids Mini-CTX-*tssA2-*Flag, Mini-CTX-*azu*-Flag, Mini-CTX-*azu*_*ss*_-Flag, Mini-CTX-*oprC*-Flag, Mini-CTX-*PA0943*-Flag, Mini-CTX-*xcpP*-Flag, Mini-CTX-*ctpA*-Flag, Mini-CTX-*cueR*, and Mini-CTX-*oprC* were constructed by a similar strategy.

Plasmid pMMB67H-*vgrG2b*-Flag was constructed by PCR amplification using the primers pMMB67H-*vgrG2b*-flag-S/A (S4 Table). The PCR products were digested with the indicated enzymes and cloned into pMMB67H. The pMMB67H-*vgrG2b*-Flag, pMMB67H-*pldA*-Flag, and pMMB67H-*oprC*-His_6_ were constructed in a similar manner.

The *clpV2* site-mutated plasmid p-*ClpV2m* was constructed using the FastaTm Fast Mutagenesis Kit (SBS Genetech) according to the manufacturer’s protocol using the primers *ClpV2*^*E286A*^-A/S and *ClpV2*^*E692A*^-A/S.

### Construction of *P*. *aeruginosa* deletion mutants

To obtain the *P*. *aeruginosa retS* deletion mutant, a SacB-based strategy was employed, as described previously [49]. Briefly, two DNA regions upstream (1980 bp) and downstream (1987 bp) of *retS* gene were amplified by PCR using the specific primer pairs pEX-*retS*-up-S/A and pEX-*retS*-down-S/A (S4 Table). The two PCR products were digested with appropriate restriction enzymes and directionally cloned into pEX18Ap [49]. The plasmids were electroporated into PAO1 with susceptibility to carbenicillin. Colonies showing both carbenicillin susceptibility and sucrose (15%) resistance were selected on LB agar plates, indicative of a double-crossover event, and thus the occurrence of gene replacement. Deletion events were verified by PCR and DNA sequencing analysis. The Δ*clpV2*, Δ*azu*, Δ*cueR*, and Δ*oprC* mutants were constructed by similar methods with the plasmids pEX-*clpV2*, pEX-*azu*, pEX-*cueR*, and pEX-*oprC*, respectively.

To make the Δ*retS*Δ*clpV2* double deletion mutant, the plasmid pEX-*clpV2* was electroporated into the Δ*retS* strain. Colonies were screened for loss of sucrose sensitivity (15%). The Δ*retS*Δ*clpV2* mutant was confirmed by PCR. The Δ*retS*Δ*clpV1*, Δ*retS*Δ*clpV3*, Δ*retS*Δ*hcp2*, Δ*retS*Δ*tssM2*, Δ*retS*Δ*vgrG2a*, Δ*retS*Δ*vgrG2b*, Δ*retS*Δ*clpV1*Δ*clpV2*Δ*clpV3* and Δ*cueR*Δ*clpV2* mutants were constructed by similar means using the plasmids listed in S3 Table.

### Secretome analysis

Secretome was performed as described previously, with minor modifications [50]. Cells were grown to an optical density at 600 nm (OD_600_) of 1.0 in M9 medium. The supernatant and pellet were separated by centrifugation. The supernatant was filtered through a 0.2-μm filter (Millipore, Billerica, MA, USA). For each 50 mL of culture supernatant, 10 mL of 100% trichloroacetic acid was added, and the fractions were incubated on ice for 4 h before centrifugation at 15,000 *g* and 4°C for 15 min. The resulting protein pellet was washed with ice-cold acetone three times. The protein pellets were re-suspended in SDS protein sample buffer and incubated at 100°C for 5 min. Samples were then resolved by 12% SDS-PAGE, and each sample was processed into eight gel bands and subjected to in-gel trypsin digestion. The resulting tryptic peptides were extracted from the gel by equilibrating the samples with 50% acetonitrile and 5% formic acid for 20 min at 37°C. Finally, peptide samples were vacuum dried and reconstituted in high-performance liquid chromatography-grade water prior to LC-MS/MS analyses, as previously described [51].

Raw MS files were processed using Proteome Discoverer (ver. 1.4.0.288; Thermo Scientific) and queried against the *P*. *aeruginosa* PAO1 protein database (downloaded from http://www.*pseudomonas*.com) using Mascot (ver. 2.3.02; Matrix Science Inc.). Monoisotopic mass was selected with a precursor mass tolerance of 20 ppm and a fragment mass tolerance of 0.8 Da. Maximum missed cleavage was set to 2. Cysteine carbamidomethylation was set as a fixed modification, and methionine oxidation was set as a variable modification. Individual proteins were considered identified when two or more unique peptides from a given protein were confirmed by ProteinProphet (cutoff score ≥0.9). The resulting assignments were filtered to achieve a false-discovery rate of <1% at both the peptide and protein levels using the target-decoy method.

The relative protein abundances from different samples were assessed using a spectral counting method. Spectral counts represent the total number of repeated identifications of peptides for a given protein during the entire analysis and provide a semiquantitative measurement of protein abundance. A normalization criterion was applied to normalize the spectral counts so that the values of the total spectral counts per sample were similar. Spectral counts were generated for each sample. Ratios of the spectral counts were calculated to identify down-regulated proteins. Only proteins with a spectral count of one or greater were considered in our analyses. Two biological replicates were performed for the secretome, which were named as group A and group B.

### Subcellular fractionation

Subcellular fractionation assays were performed according to described methods[15, 31]. *P*. *aeruginosa* strains expression indicate Flag probed proteins were grown overnight and 100-fold sub-inoculated into LB. Cells were grown to an OD_600_ of 1.0, 1 ml of cell culture were harvested and resuspended in SDS sample buffer; this sample was defined as total cell (Total). Another 5 mL of cell culture was harvested, and resuspended in 500 μL of 20 mM PBS, pH 7.3, 20% sucrose, 2.5 mM EDTA. After 20 min incubation at room temperature, 500 μL ice-cold ddH_2_O was added and gently shaken for 5 min. The suspension was centrifuged (7,000 g for 20 min at 4°C) to collect the supernatant which containing periplasmic proteins. The pellet was resuspended in SDS sample buffer; this sample was defined as cytoplasmic (Cyto). For each of supernatant, trichloroacetic acid was added and incubated on ice for 1 h before centrifugation. The resulting protein pellet was washed with ice-cold acetone. The supernatant protein pellets were re-suspended in SDS sample buffer, this sample was defined as periplasmic (Peri). Antibody against RNA polymerase α (α-RNAP) and β-lactamase (β-lac) was used as a loading control for cell cytoplasmic and periplasmic fractions, respectively.

### Secretion and western blot analyses

Overnight bacterial cultures were subcultured with 100-fold dilution in fresh LB to an OD_600_ of 3.0, or 50-fold dilution in M9 medium to an OD_600_ of 1.0. The supernatant and pellet were separated by centrifugation. The supernatant was filtered through a 0.2-μm PES filter (Millipore, Billerica, MA, USA). For each 1 mL of culture supernatant, 180 μL of 100% trichloroacetic acid was added, and the fractions were incubated on ice for 4 h before centrifugation at 15,000 *g* and 4°C for 15 min. The resulting protein pellet was washed with ice-cold acetone three times. The protein pellets were re-suspended in SDS protein sample buffer and incubated at 100°C for 10 min. For western blot analysis, 1 mL of culture was centrifuged and the total proteins were prepared by dissolving the cell pellet in 20 μL of SDS sample buffer. All samples were normalized to the OD_600_ of the culture and volume used in preparation.

For western blots, samples resolved by SDS-PAGE were transferred onto PVDF membranes. After blocking with 5% milk for 1 h at room temperature, membranes were probed with the appropriate primary antibody for 2 h at room temperature. The blots were washed several times in TBST buffer (10 mM Tris, 150 mM NaCl, 0.05% Tween) and then incubated with horseradish peroxidase-conjugated secondary antibodies in TBST buffer for 1 h. The proteins were visualized using the ECL Plus Kit (GE Healthcare, Piscataway, NJ, USA) following the manufacturer’s protocol. Antibody against RNA polymerase α (α-RNAP) was used as a loading control for cell cytoplasmic fractions.

### RNA extraction and quantitative real-time PCR

Overnight cultures of bacteria were diluted 1:100 with fresh LB broth and cells were collected. Total RNA was isolated using the RNAprep Pure Kit (For Cell/Bacteria; Tiangen Biotech, Beijing, China). cDNA was synthesized from each RNA sample using PrimeScript Reverse Transcriptase (TaKaRa) with random primers, then subjected to quantitative real-time PCR using SYBR Premix Ex Taq II (TaKaRa). The 30S ribosomal protein gene *rpsL* was used as an internal control.

### Protein expression and purification

Plasmids encoding the full-length *azu* and *cueR* genes were constructed and transferred into *E*. *coli* strains BL21(DE3), respectively. The plasmids encoding *azu* and *cueR* were constructed as follows. Genomic DNA was utilized as the template for PCR amplification with the primers *azu*-A/S and *cueR*-A/S; the PCR products were resolved on agarose gels and extracted according to the protocol. The samples were then cleaved with *Bam*HI and *Xho*I (for *azu*) or *Sal*I (for *cueR*), and the target fragments were recovered and cloned into the pGEX-6p-1 vector. The recombinant plasmids were then transferred into *E*. *coli* BL21(DE3) competent cells.

The recombinant strains were cultured in LB medium supplemented with 50 μg/mL carbenicillin at 37°C overnight. Then, 10 mL of revived bacterium suspension was inoculated into 1 L of LB medium and cultured at 37°C with continuous shaking at 200 rpm. Overexpression of the proteins was induced at an OD_600_ of 0.6 by addition of 1.0 mM isopropyl β-D-1-thiogalactopyranoside (IPTG). The induced cultures were then grown at 16°C for an additional 16 h.

Pellets were re-suspended and lysed in Buffer A [50 mM Tris-HCl (pH 7.5), 150 mM NaCl, and 10 mM PMSF]. The cells were lysed by sonication and then centrifuged at 10,000 g for 60 min. The supernatant was filtered through a 0.45-μm filter and applied to a GSTrap HP column (Qiagen). The proteins were then further purified on a HiLoad 16/60 Superdex 200 column (GE Healthcare) with Buffer C [50 mM Tris-HCl (pH 7.5), 150 mM NaCl]. The samples were concentrated to 2 mg/mL with Gel Filtration buffer and stored at stored at -80°C until use.

To obtain the non-tagged CueR protein, the GST tag was removed by incubation with PreScission Protease (GE Healthcare) for 20 h at 16°C, and untagged proteins were eluted from the GSTrap HP column with Buffer C according to the protocol.

To express and obtain the refolded recombinant OprC proteins, pET28a-*oprC* was transformed into *E*. *coli* BL21(DE3) competent cells. The *E*. *coli* cells were grown in LB to an OD_600_ of 0.5, after which 1 mM IPTG was added and growth was continued for 12 h at 26°C. OprC accumulated in inclusion bodies, which were isolated as described previously [52, 53]. The inclusion bodies were dissolved in 10 mM Tris-HCl, 150 mM NaCl, 10% glycerol, and 6 M urea (pH 7.5), and residual membranes were removed by centrifugation for 2 h at 11,000 *g*. The protein was purified on a Ni-NTA column (Qiagen) according to the manufacturer’s instructions, and then refolded into its native conformation by diluting this stock solution 20-fold in refolding buffer containing 55 mM Tris-HCl, 0.21 mM NaCl, 0.88 mM KCl, 880 mM L-arginine, and 0.5% 3-dimethyldodecylammoniopropane-sulfonate (SB-12), pH 7.0. After refolding overnight, the sample was dialyzed in 55 mM Tris-HCl, 0.21 mM NaCl, 10 mM L-arginine, and 0.5% SB-12, pH 6.5. The samples were concentrated and stored at -80°C until use.

### Electrophoretic mobility shift assay

CueR proteins were mixed with DNA probes (S4 Table) in 20 μL of gel shift buffer (25 mM HEPES, 100 mM KCl, 20 ug/mL bovine serum albumin, 3 ug/mL sspDNA, and 10% glycerol for p*tssA2* and p*hcp2*; 10 mM Tris-HCl, pH 7.5, 100 mM NaCl, 1 mM dithiothreitol, and 10% glycerol for *poprC*). After incubation at 25°C for 20 min, aliquots of binding reaction mixtures were electrophoresed on non-denaturing 6% acrylamide gels in 0.5× Tris-borate-EDTA buffer. The gels were stained with SYBR Gold dye (TransGen Biotech).

### GST Pull-down and co-immunoprecipitation assay

To obtain the Azu protein receptors, a GST pull-down assay with cell lysates was performed as described previously [54]. Briefly, 150 μg of purified GST or an equal amount GST-Azu was mixed with 150 μL of pre-washed glutathione bead slurry (Progma) in GST binding buffer (50 mM Tris, 150 mM NaCl, pH 8.0) and incubated for 2 h at 4°C, after which unbound GST proteins were washed away. Then, the wild-type PAO1 cell lysates were incubated with the protein-binding beads for additional 4 h at 4°C. The beads were then washed five times with GST washing buffer (50 mM Tris, 500 mM NaCl, pH 8.0) and bound proteins were dissolved in SDS loading buffer. After SDS-PAGE, proteins were visualized by silver staining (Bio-Rad). Individual protein bands retained by beads coated with GST-Azu, but not GST alone, were excised and analyzed by LC-MS/MS.

The *in vivo* co-immunoprecipitation assay was performed as described previously [15]. The His_6_-tagged OprC protein on pMMB67H -*oprC*-His_6_ and Flag-tagged GacA or Azu protein on Mini-CTX-*gacA*-Flag/Mini-CTX-*azu*-Flag were co-expressed in PAO1. After initial growth to an OD_600_ of 0.6, 1.0 mM IPTG was added for 8 h of induction at 37°C. Bacterial pellets were collected and re-suspended in IP lysis buffer (20 mM Tris, 150 mM NaCl, 0.1% Triton X-100, pH 8.0) containing a protease inhibitor. Cells were sonicated and centrifuged at 15,000 rpm for 30 min at 4°C to remove cell debris. Supernatants were then incubated with anti-Flag M2 magnetic beads (Sigma) for 4 h at 4°C. The beads were then washed five times with IP washing buffer (20 mM Tris, 500 mM NaCl, 0.1% Triton X-100, pH 8.0). The beads retained proteins were detected by immunoblot analysis after SDS-PAGE.

For GST pull-down experiments, 100 μg of purified GST or an equal amount GST-Azu was mixed with 50 μL of pre-washed glutathione bead slurry for 2 h at 4°C. The unbound proteins were washed away with GST binding buffer. After adding 20 μg of His_6_-OprC, binding was allowed for 4 h at 4°C. The beads were then washed five times with GST binding buffer containing 500 mM NaCl. Retained proteins were detected by immunoblot analysis after SDS-PAGE.

### Bacterial growth competition assays

Interbacterial competition assays were performed as described previously, with minor modifications [44]. Briefly, overnight cultures of relevant *P*. *aeruginosa* strains (with carbenicillin resistance) and the *E*. *coli* competitor strain containing pBBR1-MCS5 vector (conferring gentamicin resistance) were washed three times with fresh LB, and then mixed 1:1. The mixtures were 1% diluted in LB or LB containing 0.25 mM EDTA with or without 0.1 mM CuSO_4_. To calculate the initial colony-forming unit (CFU) ratio of relevant *P*. *aeruginosa* strains and competitor, 50 μL of the mixture was collected, serially diluted, spread on LB plates containing appropriate antibiotics, and incubated at 37°C for 16 h. For growth competition, the culture was incubated at 37°C for 6 h, and the mixture was serially diluted, spread on LB plates containing appropriate antibiotics, and the final CFU ratio was determined.

For intrabacterial competition assays, overnight *P*. *aeruginosa* derivative strains and the *P*. *aeruginosa* wild-type PAO1 or Δ*oprC* competitor strain containing pUCp20 vector (conferring carbenicillin resistance) were washed with fresh LB before mixing for competition. The initial *P*. *aeruginosa*/competitor ratio was 1:1 and bacteria were co-cultured in LB medium supplemented with 0.25 mM EDTA at 37°C for 6 h. After competition, the *P*. *aeruginosa* and competitor colonies were counted on LB plates containing appropriate antibiotics, and changes in the *P*. *aeruginosa*/competitor ratios were determined.

### Bacterial cell killing assay

For killing assays using *E*. *coli* as the prey, overnight cultures of relevant *P*. *aeruginosa* and prey cells were washed three times with fresh LB. Then *P*. *aeruginosa* was diluted to OD_600_ = 2.0 and prey cells to OD_600_ = 0.4 with fresh LB, the two bacteria were mixed 1:1 and 5 μL of the mixtures were co-incubated on 0.22-μm nitrocellulose membranes placed on LB agar plates. After incubation at 37°C for 12 h, the bacteria were re-suspended in 500 μL of LB and a series of 10-fold dilutions were plated on LB agar with gentamicin to select for the recipient cells harboring pBBR1-MCS5 plasmid, or LB agar with carbenicillin for recovery of *P*. *aeruginosa* and prey cells. The number of *E*. *coli* was calculated by plate CFU counts after incubation at 37°C for 16 h.

### Fluorescence microscopy

Overnight strains harboring Mini-CTX-*clpV2-sfGFP* vector for expression of the ClpV2-sfGFP fusion protein were diluted to an OD_600_ of 1.0 with fresh LB medium and grown at 37°C for 1 h. The cells were then harvested, re-suspended in PBS, and were placed on 1% agarose pads and examined immediately at room temperature. A Nikon Ti2-E inverted microscope with a perfect focus system and a CFI Plan Apo Lambda × 100 oil Ph3 DM (NA 1.4) objective lens were used for imaging. Intensilight C-HGFIE (Nikon), ET-GFP (49002, Chroma) filter sets were used to excite and filter fluorescence. NIS-Elements AR 5.20.00 was used to record and manipulate the images.

### Determination of intracellular ion content

Intracellular ion content was determined as described previously [45]. Briefly, overnight bacterial cultures were diluted 1:100 with fresh M9 medium supplemented with 1.0 mM EDTA and grown to an OD_600_ of 1.0. Next, 40-mL culture suspensions were collected, pelleted, and washed twice with PBS, and the cell pellets were chemically lysed using BugBuster (Novagen, Madison, WI, USA) according to the manufacturer’s instructions. Bacteria were re-suspended in BugBuster solution by pipetting, and incubated on a rotating mixer at a slow setting for 20 min. Total protein for each sample was measured by using a NanoDrop ND-1000 spectrophotometer (NanoDrop Technologies) according to the manufacturer’s instructions. Each sample was diluted 100-fold in 2% molecular grade nitric acid to a total volume of 6 mL. Samples were analyzed by ICP-MS (Varian 802-MS), and the results were corrected using the appropriate buffers for reference and dilution factors.

### Mouse experiment

Overnight bacterial cultures were diluted 1:100 with fresh LB medium and grown to an OD_600_ of 0.6. Six-week-old female C57BL6 mice were purchased from Harlan Laboratories (Indianapolis, IN, USA). Then, 1×10^7^ CFUs of *P*. *aeruginosa* were intranasally instilled into mice and survival rates were calculated for each bacterial strain. At 12-h post infection, mice were sacrificed by inhalation of CO_2_. Serum and lungs were isolated and homogenized in 1% proteose peptone, and CFU counts were determined by serial dilution and plating.

## Supporting information

S1 Table Proteins identified in the supernatant of PAO1, Δ*retS* and Δ*retS*Δ*clpV2* strains. (PDF)

S2 Table Azu binding proteins were identified by pull-down assays. (PDF)

S3 Table Bacterial strains and plasmids used in this study. (PDF)

S4 Table Primers used in this study. (PDF)

S1 Fig Western blot analysis using anti-Flag antibody indicates that deletion of *retS* increases the levels of Hcp2-Flag **(A)**, PldA-Flag **(B)**, and VgrG2b-Flag **(C)**. Cell lysates (Cell) and concentrated supernatant (Sup) protein fractions from the indicated strains containing Mini-CTX-hcp2-Flag **(A)** cultured in LB broth, pMMB67H-pldA-Flag **(B)** and pMMB67H-vgrG2b-Flag **(C)** cultured in M9 minimal medium were prepared and proteins were detected by western blot. For the pellet fraction, an antibody against RNA polymerase α (α-RNAP) was used as a loading control in this and subsequent blots.
(TIF)

S2 Fig Azu secretion is independent on the N-terminal signal peptide. A mini-CTX plasmid directing the expression of wild type *azu* or lacking the N-terminal signal peptide *azu*(*azu*_*ss*_) were integrated into the *P*. *aeruginosa* derivative strains, respectively. Western blot analysis of Azu_ss_-Flag or Azu-Flag in the cell-associated (Cell) and concentrated supernatant (Sup) protein fractions from the indicated strains grown in LB.
(TIF)

S3 Fig Secretion of Azu is dependent on H2-T6SS but not H1- and H3-T6SS. **(A)** The ClpV2 E286/E692 (ClpV2m) is required for Azu secretion. **(B)** Secretion of Azu is dependent on Hcp2 and TssM2. **(C)** H1- and H3-T6SS did not affect the *azu* activity. **(A-C)**, cell lysates (Cell) and concentrated supernatant (Sup) protein fractions from the indicated strains were separated by SDS/PAGE and proteins were detected by western blot. EV represents the empty vector pAK1900. **(D)** ICP-MS assays showed that mutation of *azu* or *clpV2* reduced the intracellular Cu^2+^ levels. Strains were cultured at OD_600_ = 1.0 in M9 medium containing 1.0 mM EDTA. Cu^2+^ associated with bacterial cells was measured by ICP-MS. Error bars indicate the mean ± s.d. of three biological replicates, and significance was determined by Student′s t-test: ***P<0.001.
(TIF)

S4 Fig The activity of *azu* is induced by low Cu^2+^ and repressed by high Cu^2+^. **(A)** The expression of *hcp2* and *tssA2* was induced by low Cu^2+^, but not Zn^2+^ or Ca^2+^. A mini-CTX plasmid directing the expression of Hcp2-Flag or TssA2-Flag chimera was integrated into the *P*. *aeruginosa* background strain. Bacteria were cultured in LB medium supplemented with either 0.25 mM EDTA or 0.25 mM EDTA with 0.1 mM of ZnSO_4_, CaSO_4_ or CuSO_4_. **(B)** Western blot analysis showed that 0.25 mM EDTA efficiently activates the *azu* expression. The activity of Azu was repressed by high Cu^2+^. **(C)** The expression of H1- and H3-T6SS was not regulated by Cu^2+^. **(A-C)** cell lysates (Cell) and concentrated supernatant (Sup) protein fractions from the indicated strains cultured in LB medium containing EDTA with or without CuSO_4_ were separated by SDS/PAGE and protein was detected by western blot assays.
(TIF)

S5 Fig The activity of H2-T6SS is induced by low Cu^2+^ and repressed by high Cu^2+^. **(A)** Cu^2+^ influences H2-T6SS assembly. Chromosomally encoded ClpV2-sfGFP localization in the *P*. *aeruginosa* measured by fluorescence microscopy. Cells were grown in the indicated conditions to OD_600_ = 1.0 and H2-T6SS activated were analyzed. N = total number of cells analyzed for each strain. Bacteria was cultured in LB medium supplemented with either 0.25 mM EDTA or 0.25 mM EDTA with 0.1 mM of ZnSO_4_, CaSO_4_ or CuSO_4_. **(B)** The stability of ClpV2-sfGFP was not influenced by EDTA, Cu^2+^, CueR, and RetS. Cell fractions were separated by SDS/PAGE and protein were detected by western blot assays.
(TIF)

S6 Fig The expression of *azu* is negatively regulated by CueR. **(A)** The expression of *azu* was examined in wild-type PAO1, the *cueR* mutant, and the Δ*cueR* complemented strain (Δ*cueR/*p*-cueR*). Data shown are the average and SD from three independent experiments. Significance was determined by Student′s *t*-test, **P<0.01. **(B)** The indicated strains containing Mini-CTX-*azu-*Flag plasmid were cultured at OD_600_ = 1.0 in LB medium. Cell lysates (Cell) and concentrated supernatant (Sup) protein fractions from the indicated strains were separated by SDS/PAGE and proteins were detected by western blot **(C)** Deletion of *cueR* increases the levels Hcp2 (upper) and TssA2 (down) relative to wild-type PAO1. Western blot analysis of Hcp2-Flag and TssA2-Flag in the cell-associated fractions from wild-type PAO1 and Δ*cueR*, strain cultured in LB medium containing 0.25 mM EDTA with or without 0.1, 0.25 and 0.5 mM CuSO_4_. Cell fractions were separated by SDS/PAGE and protein were detected by western blot assays.
(TIF)

S7 Fig CueR regulates H2-T6SS expression but does not affect the H1- and H3-T6SS activity. **(A)** The mRNA levels of *tssA2*, *clpV2*, and *hcp2* in wild-type PAO1, Δ*cueR* mutant, and its complemented strain (Δ*cueR/*p*-cueR*) was examined by qRT-PCR. Strains were cultured to an OD_600_ = 0.8 in LB broth or LB supplemented with either 0.25 mM EDTA or 0.25 mM EDTA with 0.1 mM Cu^2+^.at 37°C. Cultures were harvested, and total RNA was isolated. Error bars represented the mean ± s.d. from three independent experiments. *P<0.05, **P<0.01, ***P<0.001 based on two-way ANOVA Dunnett′s multiple comparison test. NS, not significance. EV represents the empty vector pAK1900. **(B)** Western blot assays showed that CueR did not influence Hcp1 and Hcp3 protein levels. Intracellular Hcp1 and Hcp3 proteins were separated by centrifugation. Samples were subjected to SDS/PAGE gels and probed with an anti-Flag antibody. **(C)** EMSAs showed that CueR bound to the truncated fragment of *hcp2*_-208 to -159_ and *tssA2*_-173 to -140_. PCR products were added to the reaction mixture at a concentration of 2.0 ng. The protein concentration of each sample is indicated above its lane. As a negative control, no band shift was observed when CueR protein incubates with PA1374 promoter region.
(TIF)

S8 Fig **(A)** SDS-PAGE analysis of the samples in Co-IP assay. Cell lysates of *P*. *aeruginosa* containing pMMB67H-OprC-His with either p-GacA-Flag or p-Azu-Flag were individually incubated with Flag beads, and beads retained proteins were strained by Coomassie Blue R-250. **(B)** Azu interaction with OprC. His6-OprC was incubated with GST-Azu or GST, and the protein complexes captured with glutathione beads were detected by western blot. The single asterisk and double asterisks represent GST-Azu and GST protein, respectively. Data are representative of two replications. **(C)** CueR binds to the promoter region of oprC. PCR products were added to the reaction mixtures at 2.0 ng. The PA1374 promoter region showing no binding with CueR protein as a negative control. **(D)** Protein levels of OprC were tested in wild-type PAO1, Δ*cueR* mutant, and its complemented strain (Δ*cueR*/p*-cueR*). The indicated strains containing OprC-Flag were cultured to an OD_600_ = 1.0 in LB broth at 37°C. The pellet fractions of cells were analyzed by western blot using anti-Flag antibody. EV represents the empty vector pAK1900.
(TIF)

S9 Fig **(A)** Schematics of conserved domains of OprC identified by using Pfam. Plug, the TonB-plug domain (residues 75–181); TonB-dep-Rec, the TonB-dependent receptor domain (residues 224–722). **(B)** OprC homologs are widely distributed in *Pseudomonas* species. The phylogeny generated by the neighbor-joining algorithm in MEGA 7.0 illustrates that OprC is highly conserved among the vast majority of *Pseudomonas*. The PAO1 OprC was highlighted by red. The bar represents the genetic distance.
(TIF)

S1 Movie Visualization of H2-T6SS assembly. Chromosomally encoded ClpV2-sfGFP localization in the *P*. *aeruginosa* was imaged over 10 minutes with a rate of 1 image per 20 seconds. The movie is played at a rate of 10 frames per second. Cells were grown in LB medium supplemented with either 0.25 mM EDTA or 0.25 mM EDTA with 0.1 mM CuSO_4_ to OD_600_ = 1.0 and spotted on a 0.5 × PBS agarose pad prior to imaging. Δ*retS* is a positive control.
(MP4)

S2 Movie Zn^2+^ and Ca^2+^ does not influence H2-T6SS assembly. Chromosomally encoded ClpV2-sfGFP localization in the *P*. *aeruginosa* was imaged over 10 minutes with a rate of 1 image per 20 seconds. The movie is played at a rate of 10 frames per second. Cells were grown in LB medium supplemented with either 0.25 mM EDTA or 0.25 mM EDTA with 0.1 mM ZnSO4, CaSO4 or CuSO_4_ to OD_600_ = 1.0 and spotted on a 0.5 × PBS agarose pad prior to imaging.
(MP4)

S3 Movie Deletion of *cueR* promotes H2-T6SS assembly. Chromosomally encoded ClpV2-sfGFP localization in the *P*. *aeruginosa* wild-type PAO1/EV, Δ*cueR/*EV, and the complemented strain (Δ*cueR/*p*-cueR*) were imaged over 10 minutes with a rate of 1 image per 20 seconds. The movie is played at a rate of 10 frames per second. Cells were grown in LB medium to OD_600_ = 1.0 and spotted on a 0.5 × PBS agarose pad prior to imaging.
(MP4)
