# Supplementary material for: A Pseudomonas aeruginosa type VI secretion system regulated by CueR facilitates copper acquisition
Source: PLoS Pathog. 2019 Dec 2;15(12):e1008198. doi: 10.1371/journal.ppat.1008198 (PMC6907878; doi:10.1371/journal.ppat.1008198)
Supplement: S1 Table — (PDF) [file ppat.1008198.s001.pdf]

S1 Table. Proteins identified in the supernatant of PAO1, Δ*retS* and Δ*retS*Δ*clpV2* strains.

Protein ID	Protein_Description	Group A				Group B				Average Rate[#]
		Sequence coverage			Rate* (△*retS*△*clpV2*/△*retS*)	Sequence coverage			Rate* (△*retS*△*clpV2*/△*retS*)	
		**PAO1**	**△*retS***	**△*retS*△*clpV2***		**PAO1**	**△*retS***	**△*retS*△*clpV2***		
PA0260	Tle3		75	2	0.027		25		0	0.014
PA0261	Hypothetical protein		22	2	0.091	7	56	10	0.179	0.135
PA0262	VgrG2b		118		0		155		0	0
PA0263	Secreted protein Hcp	23	512	6	0.012	13	836	37	0.044	0.028
PA0423	PasP	98	200	93	0.465	50	144	69	0.479	0.472
PA0604	AgtB		2		0	3	35	2	0.057	0.029
PA0807	AmpDh3	27	31	11	0.355	15	43	9	0.209	0.282
PA0888	Arginine/ornithine binding protein AotJ	21	67	18	0.269	37	105	30	0.286	0.278
PA1510	Tle4		46		0		12		0	0
PA1511	VgrG2a		109		0		178		0	0
PA1639	Hypothetical protein		11	3	0.273		50	22	0.440	0.357
PA1863	Molybdate-binding periplasmic protein precursor ModA	3	78	24	0.308	39	118	28	0.237	0.273
PA2328	Hypothetical protein		2		0		14	3	0.214	0.107
PA2395	PvdO	23	55	27	0.491	30	167	80	0.479	0.485
PA2464	Hypothetical protein		55	20	0.364	6	55	25	0.455	0.410
PA2592	Spermidine/putrescine-binding protein		5	2	0.400		19	8	0.421	0.411
PA2856	Lysophospholipase A		11	5	0.455	6	18	6	0.333	0.394
PA3313	Hypothetical protein	23	60	27	0.450	73	179	71	0.397	0.424
PA3487	Tle5	17	527	14	0.027	15	636	35	0.055	0.041
PA4922	Azurin precursor	14	279	81	0.290	13	201	48	0.239	0.265
PA5498	Probable adhesin		9	2	0.222	1	10	1	0.100	0.161

***: Rate(△*retS*△*clpV2*/△*retS*),**

[#]: Average Rate < 0.5
