# Supplementary material for: A Pseudomonas aeruginosa type VI secretion system regulated by CueR facilitates copper acquisition
Source: PLoS Pathog. 2019 Dec 2;15(12):e1008198. doi: 10.1371/journal.ppat.1008198 (PMC6907878; doi:10.1371/journal.ppat.1008198)
Supplement: S2 Table — (PDF) [file ppat.1008198.s002.pdf]

S2 Table. binding proteins were identified by pull-down assays.

Protein ID	Protein Description	Sequence coverage*
PA3790	Putative copper transport outer membrane porin OprC precursor	172
PA0588	Conserved hypothetical protein	31
PA1596	Heat shock protein HtpG	16
PA4265	Elongation factor Tu	12
PA0688	Low-molecular-weight alkaline phosphatase A, LapA	10

***: Sequence coverage > 10.**
