# Supplementary material for: A Pseudomonas aeruginosa type VI secretion system regulated by CueR facilitates copper acquisition
Source: PLoS Pathog. 2019 Dec 2;15(12):e1008198. doi: 10.1371/journal.ppat.1008198 (PMC6907878; doi:10.1371/journal.ppat.1008198)
Supplement: S3 Table — (PDF) [file ppat.1008198.s003.pdf]

S3 Table. Bacterial strains and plasmids used in this study.

Strain or plasmid	Relevant characteristics	Source
E. coli		
DH5α	*F^- φ80lacZ ΔM15 Δ(lacZYA-argF)U169 recA1 endA1 hsdR17(rk^-, mk^+)phoA supE44 thi-1 gyrA96 relA1 tonA*	Stratagene
BL21 (DE3)	F^- *ompT hsdS*$_B$ (rB^- m_B^-) *gal dcm met* (DE3)	Invitrogen
P. aeruginosa		
PAO1	Wild type	This lab
Δ*retS*	*retS* deletion mutant of PAO1	This lab
Δ*retS*Δ*clpV2*	*retSclpV2* deletion mutant of PAO1	This study
Δ*retS*Δ*hcp2*	*retShcp2* deletion mutant of PAO1	This study
Δ*retS*Δ*tssM2*	*retStssM2* deletion mutant of PAO1	This study
Δ*retS*Δ*vgrG2a*	*retSvgrG2a* deletion mutant of PAO1	This study
Δ*retS*Δ*vgrG2b*	*retSvgrG2b* deletion mutant of PAO1	This study
Δ*retS*Δ*clpV1*	*retSclpV1* deletion mutant of PAO1	This study
Δ*retS*Δ*clpV3*	*retSclpV3* deletion mutant of PAO1	This study
Δ*retS*Δ*clpV123*	*retSclpV1clpV2clpV3* deletion mutant of PAO1	This study
Δ*clpV2*	*clpV2* deletion mutant of PAO1	This study
Δ*azu*	*azu* deletion mutant of PAO1	This study
Δ*oprC*	*oprC* deletion mutant of PAO1	This study
Δ*cueR*	*cueR* deletion mutant of PAO1	This study
Δ*cueR*Δ*clpV2*	*cueRclpV2* deletion mutant of PAO1	This study
**Plasmids**		
pET28a	T7 *lac* promoter-operator, N-terminal His tag, Kan^r	Novagen
pGEX6p-1	Expression vector with N-terminal GST tag, Ap^r	Novagen
pEX18Ap	*$oriT^+$ $sacB^+$* gene replacement vector with multiple-cloning site from pUC18; Ap^r	1
pAK1900	*E. coli-P. aeruginosa* shuttle cloning vector carrying p*lac* upstream of MCS; Ap^r, Cb^r	2
mini-CTX-*lacZ*	Integration plasmid; Tc^r	3
pMMB67H	*E. coli-P. aeruginosa* shuttle cloning vector; Ap^r, Cb^r	4
pUCp26	*E. coli-P. aeruginosa* shuttle cloning vector; Tc^r	5
pET28a-*oprC*	Protein expression construct, the entire gene of *oprC* cloned in pET28a vector	This study
pGEX6p-1-*azu*	Protein expression construct, the entire gene of *azu* cloned in pGEX-6p-1 vector	This study
pGEX6p-1-*cueR*	Protein expression construct, the entire gene of *cueR* cloned in pGEX-6p-1 vector	This study
pEX-*clpV2*	*clpV2* deletion plasmid, pEX18Ap with upstream and downstream region of *clpV2*; Ap^r	This study
pEX-*hcp2*	*hcp2* deletion plasmid, pEX18Ap with upstream and downstream region of *hcp2*; Ap^r	This study
pEX-*tssM2*	*tssM2* deletion plasmid, pEX18Ap with upstream region and	This study

	downstream region of *tssM2*; Ap^r	
pEX-*clpV1*	*ClpV1* deletion plasmid, pEX18Ap with upstream and downstream region of *clpV1*; Ap^r	This study
pEX-*clpV3*	*clpV3* deletion plasmid, pEX18Ap with upstream and downstream region of *clpV3*; Ap^r	This study
pEX-*vgrG2a*	*VgrG2a* deletion plasmid, pEX18Ap with upstream and downstream region of *clpV3*; Ap^r	This study
pEX-*vgrG2b*	*vgrG2b* deletion plasmid, pEX18Ap with upstream and downstream region of *clpV3*; Ap^r	This study
pEX-*azu*	*azu* deletion plasmid, pEX18Ap with upstream and downstream region of *azu*; Ap^r	This study
pEX-*oprC*	*oprC* deletion plasmid, pEX18Ap with upstream and downstream of *oprC*; Ap^r	This study
mini-CTX-*oprC*	Expression plasmid, mini-CTX-*lacZ* containing the entire *oprC* gene; Tc^r	This study
mini-CTX-*hcp2*-Flag	Expression plasmid, mini-CTX-*lacZ* containing the entire *hcp2* gene and the 3x *flag* sequence; Tc^r	This study
mini-CTX-*oprC*-Flag	Expression plasmid, mini-CTX-*lacZ* containing the entire *oprC* gene and the 3x *flag* sequence; Tc^r	This study
mini-CTX-*azu*-Flag	Expression plasmid, mini-CTX-*lacZ* containing the entire *azu* gene and the 3x *flag* sequence; Tc^r	This study
mini-CTX-azu_{ss}-Flag	Expression plasmid, mini-CTX-*lacZ* containing the entire *azu* gene lack the N-terminal signal peptide and the 3x *flag* sequence; Tc^r	This study
mini-CTX-*tssA2*-Flag	Expression plasmid, mini-CTX-*lacZ* containing the entire *tssA2* gene and the 3x *flag* sequence; Tc^r	This study
mini-CTX-*PA0943*-Flag	Expression plasmid, mini-CTX-*lacZ* containing the entire *PA0943* gene and the 3x *flag* sequence; Tc^r	This study
mini-CTX-*xcpP*-Flag	Expression plasmid, mini-CTX-*lacZ* containing the entire *xcpP* gene and the 3x *flag* sequence; Tc^r	This study
mini-CTX-*ctpA*-Flag	Expression plasmid, mini-CTX-*lacZ* containing the entire *ctpA* gene and the 3x *flag* sequence; Tc^r	This study
mini-CTX-lacZ-clpV2-sfGFP	Expression plasmid, mini-CTX-*lacZ* containing the entire *clpV2* gene fused with *sfGFP* sequence; Tc^r	This study
pMMB67H-*vgrG2b-Flag*	Expression plasmid, pMMB67H containing the entire *vgrG2b* gene and the 3x *flag* sequence; Ap^r, Cb^r	This study
pMMB67H-*pldA-Flag*	Expression plasmid, pMMB67H containing the entire *pldA* gene and the 3x *flag* sequence; Ap^r, Cb^r	This study
pMMB67H-*oprC-His*$_6$	Expression plasmid, pMMB67H containing the entire oprC gene and the 3x his sequence; Ap^r, Cb^r	This study
pAK-*clpV2*	pAK1900 with the entire *clpV2* gene; Ap^r	This study
pAK-$clpV2_{2E}$	pAK1900 with the site-directed mutant *clpV2* gene; Ap^r	This study
pAK-*hcp2*	pAK1900 with the entire *hcp2* gene; Ap^r	This study

pAK-*tssM2*	pAK1900 with the entire *tssM2* gene; Ap^r	This study
pAK-*cueR*	pAK1900 with the entire *cueR* gene; Ap^r	This study
pAK-*azu*	pAK1900 with the entire *azu* gene; Ap^r	This study
pUCp26-*retS*	pUCp26 with the entire *retS* gene; Tc^r	This lab
pUCp26-*clpV2*	pUCp26 with the entire *clpV2* gene; Tc^r	This study

Reference

1. Hoang, T.T., Karkhoff-Schweizer, R.R., Kutchma, A.J. & Schweizer, H.P. A broad-host-range Flp-FRT recombination system for site-specific excision of chromosomally-located DNA sequences: application for isolation of unmarked *Pseudomonas aeruginosa* mutants. Gene 212, 77-86 (1998).
2. Poole, K., Neshat, S., Krebes, K. & Heinrichs, D.E. Cloning and nucleotide sequence analysis of the ferripyoverdine receptor gene *fpvA* of *Pseudomonas aeruginosa*. J Bacteriol 175, 4597-604 (1993).
3. Becher, A. & Schweizer, H.P. Integration-proficient *Pseudomonas aeruginosa* vectors for isolation of single-copy chromosomal *lacZ* and *lux* gene fusions. BioTechniques 29, 948-953 (2000).
4. Furste JP, Pansegrau W, Frank R, Blocker H, Scholz P, Bagdasarian M, Lanka E (1986) Molecular cloning of the plasmid RP4 primase region in a multi-host-range *tacP* expression vector. Gene 48: 119-131.
5. West SE, Schweizer HP, Dall C, Sample AK, Runyen-Janecky LJ (1994) Construction of improved *Escherichia*-*Pseudomonas* shuttle vectors derived from pUC18/19 and sequence of the region required for their replication in *Pseudomonas aeruginosa*. Gene 148: 81-86.
