# Supplementary material for: A Pseudomonas aeruginosa type VI secretion system regulated by CueR facilitates copper acquisition
Source: PLoS Pathog. 2019 Dec 2;15(12):e1008198. doi: 10.1371/journal.ppat.1008198 (PMC6907878; doi:10.1371/journal.ppat.1008198)
Supplement: S4 Table — (PDF) [file ppat.1008198.s004.pdf]

S4 Table. Primers used in this study.

Primer	Sequence (5'→3')[a]	Application
pEX-*clpV1*-up-S	ATAgaattcGACCGGGTCAACGAGCATCA	Constructing *clpV1*
pEX-*clpV1*-up-A	GACtctagaCTTGCAGAATACCGTGGCGG	deletion mutant
pEX-*clpV1*-down-S	TATtctagaCCTCACCCGCATGCTCGAAG	
pEX-*clpV1*-down-A	TATaagcttGAATTGAGGTCGAGGCGGCC	
pEX-*clpV2*-up-S	TACggatccCATCCAGCGCCTGCACCCGA	Constructing *clpV2*
pEX-*clpV2*-up-A	CGTtctagaTTCGAGGTCGCGCCGGGTGT	deletion mutant
pEX-*clpV2*-down-S	TATtctagaTTTGCTGGAAGCGATGGCGC	
pEX-*clpV2*-down-A	TATaagcttGCGATCGGGAATCGCCCAAT	
pEX-*clpV3*-up-S	TTAgaattcCATTTGCAGGCGGAAGACTT	Constructing *clpV3*
pEX-*clpV3*-up-A	TCCtctagaTAATGATGGGTGCGTTGCAG	deletion mutant
pEX-*clpV3*-down-S	TATtctagaGCCGTGCTCGCCAAGTGCAG	
pEX-*clpV3*-down-A	AATaagcttCGTCGTCATGCCCGCTGAGG	
pEX-*retS*-up-S	TTAggtaccGGGCTGGGACTTCAAGCGGG	Constructing *retS*
pEX-*retS*-up-A	GGCtctagaGATCCGAAGCCGTACCACGG	deletion mutant
pEX-*retS*-down-S	TATtctagaGGCGACGCCCTGCCCTCCTG	
pEX-*retS*-down-A	TATaagcttCGCCGCCGTCCTCGCTCATC	
pEX-*hcp2*-up-S	TATggatccCCTGGTGCTCCCCGGCGTAG	Constructing *hcp2*
pEX-*hcp2*-up-A	CGCtctagaCCTCCGGCTCCGACGACTGG	deletion mutant
pEX-*hcp2*-down-S	ATAtctagaCTTGGTGCCGGTGATGGACA	
pEX-*hcp2*-down-A	TATaagcttCATCCGGTGCTGACGATCGA	
pEX-*tssM2*-up-S	TTAcccgggCAATACCTCGAACGTGCC	Constructing *tssM2*
pEX-*tssM2*-up-A	GCCtctagaCATTTCGCGTCCCTGTGT	deletion mutant
pEX-*tssM2*-down-S	GCCtctagaCTCTGATGCAACGCACCG	
pEX-*tssM2*-down-A	ATTaagcttACGGTGCCAGCGGCGCAC	
pEX-*vgrG2a*-up-S	TATgaattcGATCAACCCTGGCTGCCAGT	Constructing *vgrG2a*
pEX-*vgrG2a*-up-A	CAGtctagaGCGACACGGAGTCCAACACA	deletion mutant
pEX-*vgrG2a*-down-S	TAAtctagaGAGTTTCCCTTCGCCGACGA	
pEX-*vgrG2a*-down-A	TATaagcttCGGCAACGTGTTCAGGGTGA	
pEX-*vgrG2b*-up-S	ATAggatccGTGACGGCTCCGTCGAAATC	Constructing *vgrG2b*
pEX-*vgrG2b*-up-A	TCGtctagaAGCAGGTTCGCTGGCGTACT	deletion mutant
pEX-*vgrG2b*-down-S	TCAtctagaGAGTTTCCCTTCGCCGACGA	
pEX-*vgrG2b*-down-A	TCTaagcttTCTCATGGGTGCATGGTGGG	
pEX-*cueR*-up-S	TTAgaattcGCTCACCCCGGTGCTGCT	Constructing *cueR*
pEX-*cueR*-up-A	CCCtctagaCGCTTCACCGATGTTCAT	deletion mutant
pEX-*cueR*-down-S	ATGtctagaGCGTCTGGCTGCTGCCAC	
pEX-*cueR*-down-A	ATTaagcttCGCTTTCCTGATCCGCCAGA	
pEX-*oprC*-up-S	TATgaattcGAGAAGTGAGCAACGCGGCG	Constructing *oprC*
pEX-*oprC*-up-A	CGAtctagaCTGAACCTTCAACGAGGCCC	deletion mutant
pEX-*oprC*-down-S	TAAtctagaCATGATCTATGTGACTTCCGCCC	
pEX-*oprC*-down-A	TATctgcagCGATGTCGGAGAAGTCCAGTTG	
pEX-*auz*-up-S	ACTgaattcCCTAACGGGAGCCCGAACAT	Constructing *auz*
pEX-*auz*-up-A	CTGtctagaACCCTGAAGTGATGCGCGAG	deletion mutant

pEX-*auz*-down-S	GTGtctagaCGTAGCATGGAGCAGCCTCCT	
pEX-*auz*-down-A	CAGaagcttAGCCGCCGGTGGACG	
pET-*oprC*-S	TATggatccATGGAAAAACGTATGTCTACTCA	Protein cloning
pET-*oprC*-A	TATctcgagTCAGAAGCTGAAGTCGACCTTGG	
pGEX6p-1-*auz*-S	GTAggatccATGCTACGTAAACTCGCTGCG	Protein cloning
pGEX6p-1-*auz*-A	GATctcgagTCACTTCAGGGTCAGGGTGCC	
pGEX6p-1-*cueR*-S	ATAggatccATGAACATCGGTGAAGCGG	Protein cloning
pGEX6p-1-*cueR*-A	ATAgtcgacTCAGTGGCAGCAGCCAGAC	
pAK-*clpV2*-S	TATtctagaGGGGTATGTGGGGATGCTGGAT	Constructing *clpV2*
pAK-*clpV2*-*overlap*-A	TACGTTGATCATGCTCATCCTAACCCTTCA	complemented
pAK-*clpV2*-*overlap*-S	CATTGAAGGGTTAGGATGAGCATGATCAA	plasmid
pAK-*clpV2*-A	CATaagcttTGCGGTACGCGGCTGAACAT	
pAK-*hcp2*-S	TTAtctagaTGTACGACCGGTGTGCCTGT	Constructing *hcp2*
pAK-*hcp2*-A	ACTaagcttACCTGATCGGCAGCTGGATC	complemented plasmid
pAK-*tssM2*-S	TTAtctagaATGCCTGGATGGCAGCGG	Constructing *tssM2*
pAK-*tssM2*-*overlap*-A	GAAGAAGTTTTTCATCGAGTGGCTGAGTTC	complemented
pAK-*tssM2*-*overlap*-S	GAACTCAGCCACTCGATGAAAAACTTCTTC AAGAAAGTCGGCG	plasmid
pAK-*tssM2*-A	ATCaagcttAGGGCTCGGTGCGTTGCATCAGA	
pAK-*cueR*-S	TAAggatccGATCGTCAAGCGCATCGTCG	Constructing *cueR*
pAK-*cueR*-A	TCAaagcttCTGGCCGCTGCTGGATATCA	complemented plasmid
pAK-*auz*-S	ATAtctagaGATCGACTCCTGTCGGGGG	Constructing *auz*
pAK-*auz*-A	TATaagcttTCACTTCAGGGTCAGGGTGCC	complemented plasmid
Mini-*oprC*-S	ATAgaattcAGGCAGGCGTGTTGGCGG	Constructing *oprC*
Mini-*oprC*-overlap-A	CTGCGACTCCCGCCGTC	complemented
Mini-*oprC*-overlap-S	GACGGCGGGAGTCGCAGATGGAAAAACGT ATGTCTACTCAAC	plasmid & Western-blot
Mini-*oprC*-A	TATctcgagTCAGAAGCTGAAGTCGACCTTGG	
Mini-*oprC*-flag-A	TATgaattcGAAGCTGAAGTCGACCTTGGTCC	
Mini-*hcp2*-flag-S	TATctcgagGCCTTGGCCGAAAGGCTA	Western-blot
Mini-*hcp2*-flag-A	TATgaattcCCCTGCCACCGGCGA	
Mini-*auz*-flag-S	ATActcgagGATCGACTCCTGTCGGGG	Western-blot
Mini-auz_{ss}-flag-overlap-A	CATGGAGCAGCCTCCTA	
Mini-auz_{ss}-flag-overlap-S	TAGGAGGCTGCTCCATGCCGAGTGCTCGGT	
Mini-*auz*-flag-A	TATgaattcCTTCAGGGTCAGGGTGCC	
Mini-*tssA2*-flag-S	TATgtcgacGGGGAGCTTCGATCAGCG	Western-blot
Mini-*tssA2*-flag-A	TATaagcttTTCGAGCACCACCTCGAGATC	
Mini-*xcpP*-S	CGGggtaccCAGCCAGTACTGCTCCTCCAG	Western-blot
Mini-*xcpP*-A	CCCaagcttGTCGCTTTCCGTAGGCGTG	
Mini-*PA0943*-S	CGGggtaccAGCAGGGTCTGCAGGGCT	Western-blot
Mini-*PA0943*-overlap-A	AAATCCAGGCCTATAGGGGTAAT	

Mini-*PA0943*-overlap-S	AATTACCCCTATAGGCCTGGATTTATGCGTAAAGCCATGCTGTTC	
Mini-*PA0943*-A	CCCaagcttGCTCTTTTCGCCAGTGACGG	
Mini-*ctpA*-S	CGGggtaccCGCTGCTGATGCTGTTCGC	Western-blot
Mini-*cptA*-overlap-A	GGGGTGGCGGGCACCTGG	
Mini-*cptA*-overlap-S	CCAGGTGCCCGCCACCCCATGCTGCATTGCTTCCGTCC	
Mini-*cptA*-A	CCCaagcttGTTGCCGCGGGTGACGCT	
Mini-*clpV2-sfGFP*-S	CCCaagcttGGTATGTGGGGATGCTGGAT	Fluorescence microscopy & Western-blot
Mini-*clpV2-sfGFP*-overlap-A	AACAGTTCTTCACCTTTACAGGCCAACTCGCAGAG	
Mini-*clpV2-sfGFP*-overlap-S	CTCTGCGAGTTCGCCTCTAAAGGTGAAGAACTGTT	
Mini-*clpV2-sfGFP*-flag-A	AGTgaattcTTATTTGTAGAGCTCATCCATGC	
pMMB67H-*vgrG2b*-flag-S	CCGgaattcATGCGTCAAAGGGACCTGAA	Western-blot
pMMB67H-*vgrG2b*-flag-A	CCCaagcttGTATCCCGTTGGGAAGTTTTTC	
pMMB67H-*pldA*-flag-S	ATAgaattcATGTTGCAGAAGAAGCCCTACAA	Western-blot
pMMB67H-*pldA*-flag-A	TATaagcttTGTGCGATGCTTTCCTTGTGC	
pMMB67H-*oprC-His-S*	CGCgaattcATGGAAAAACGTATGTCT	Co-IP
pMMB67H-*oprC-His-A*	AATaagcttTCAGTGATGATGATGATGATGGAAGCTGAAGTCGAC	
clpV2^{E286A}-S	ACCATCCTGTTCATCGACGCGGCCCATACGCTGAT	Mutagenesis
clpV2^{E286A}-A	ATCAGCGTATGGGCCGCGTCGATGAACAGGATGGT	
clpV2^{E692A}-S	TTCGGTGATATTGCTGGACGCGGTGGAGAAGGCCGATC	
clpV2^{E692A}-A	GATCGGCCTTCTCCACCGCGTCCAGCAATATCACCGAA	
qRT-*rpsL*-S	GTAAGGTATGCCGTGTACG	For qRT PCR
qRT-*rpsL*-A	CACTACGCTGTGCTCTTG	
qRT-*oprC*-S	CGCAGTCCGACGATTACGA	
qRT-*oprC*-A	GGCGGTGAGTTCGATCAGG	
qRT-*azu*-S	TGTCGTCGGGCTTCAGG	
qRT-*azu*-A	CAACACCAATGCCATCACC	
qRT-*clpV2*-S	GATCCGTGGCCAGGAAC	
qRT-*clpV2*-A	CACCAGCAGGAACACGC	
qRT-*hcp2*-S	CACTACTACACCACCGTCCT	
qRT-*hcp2*-A	GTGGGTCCAGGTGATCTTGC	
qRT-*tssA2*-S	CCCATCGAGTCCATGCCC	
qRT-*tssA2*-A	CGCTGATCCAGCCACGGG	
qRT-*tssF2*-S	GGTGGGGTTCGCCGAAGA	
qRT-*tssF2*-A	GCTGTATTCGGCCGGCAT	

qRT-*hcp1*-S	CCAAGTACATCGACAAGT	
qRT-*hcp1*-A	TTCCTTCAGGGTGATGAT	
qRT-*hcp3*-S	AAGGCGACAGCCTGCTCG	
qRT-*hcp3*-A	CAACTGGTTGGCGGCCAG	
phcp2-gf-S	GCCTTGGCCGAAAGGCTA	For gel-shift
phcp2(208)-gf-S	TCCGAGAGAGTGCGCAACTTT	
phcp2(158)-gf-S	TTTTCAGTGTTCTTCCCATGAG	
phcp2-gf-A	GGATACGTTCCTTGTCGTTGAAAG	
ptssA2-gf-S	GGACCGCCCATCTCGATT	
ptssA2(173)-gf-S	AATAGGAAAATTCCCAAAGA	
ptssA2(139)-gf-S	GCTTGCCAGTTTTTATGTTG	
ptssA2-gf-A	CCTAACCCTTCAATGCACACC	
poprC-gf-S	AGGCAGGCGTGTTGGCGG	
poprC-gf-A	CTGCGACTCCCGCCGTCT	
p1374-gf-S	GACCTTCGCCGATGCCGA	
p1374-gr-A	AGCCGGTAGCTCGCTCCG	

[a] Restriction sites displayed in lowercase.
