# Supplementary figures and images for: A Pseudomonas aeruginosa type VI secretion system regulated by CueR facilitates copper acquisition
Source: PLoS Pathog. 2019 Dec 2;15(12):e1008198. doi: 10.1371/journal.ppat.1008198 (PMC6907878; doi:10.1371/journal.ppat.1008198)

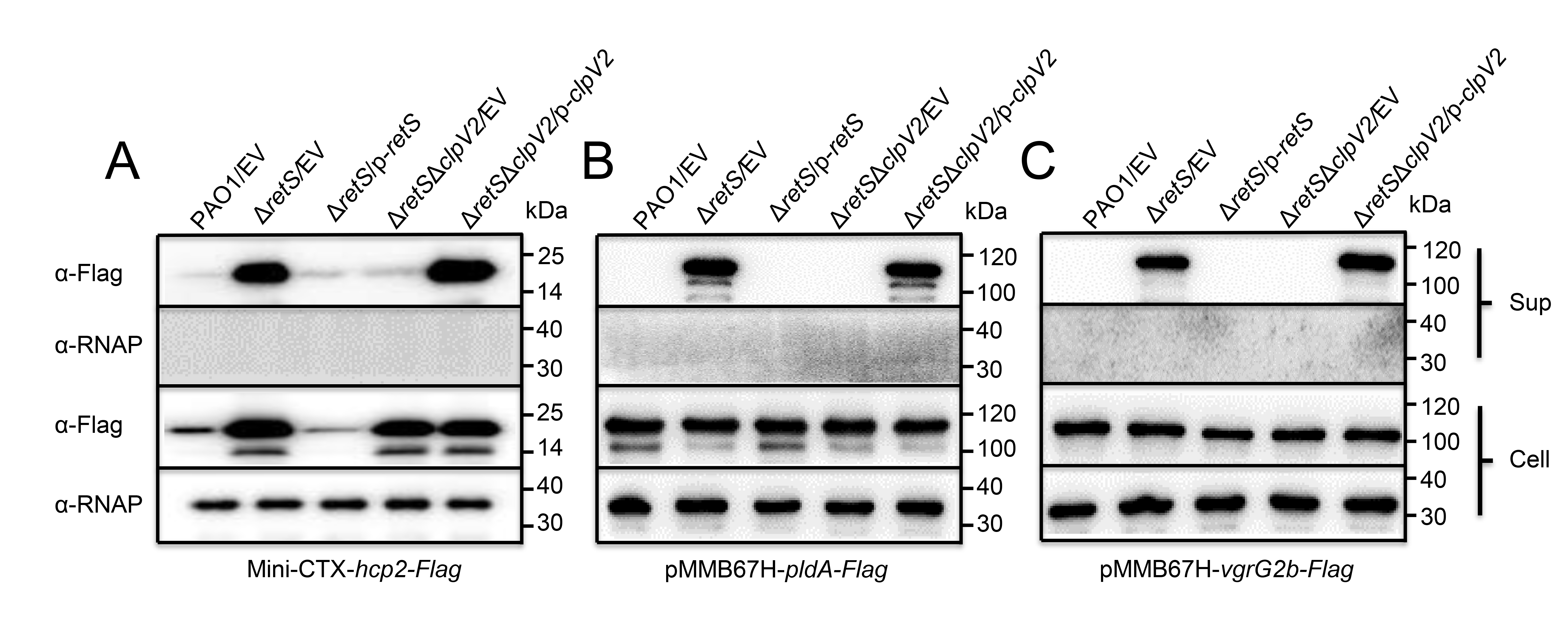

Supplement: S1 Fig — Western blot analysis using anti-Flag antibody indicates that deletion of retS increases the levels of Hcp2-Flag (A), PldA-Flag (B), and VgrG2b-Flag (C). Cell lysates (Cell) and concentrated supernatant (Sup) protein fractions from the indicated strains containing Mini-CTX-hcp2-Flag (A) cultured in LB broth, pMMB67H-pldA-Flag (B) and pMMB67H-vgrG2b-Flag (C) cultured in M9 minimal medium were prepared and proteins were detected by western blot. For the pellet fraction, an antibody against RNA polymerase α (α-RNAP) was used as a loading control in this and subsequent blots. (TIF) [file ppat.1008198.s005.tif]

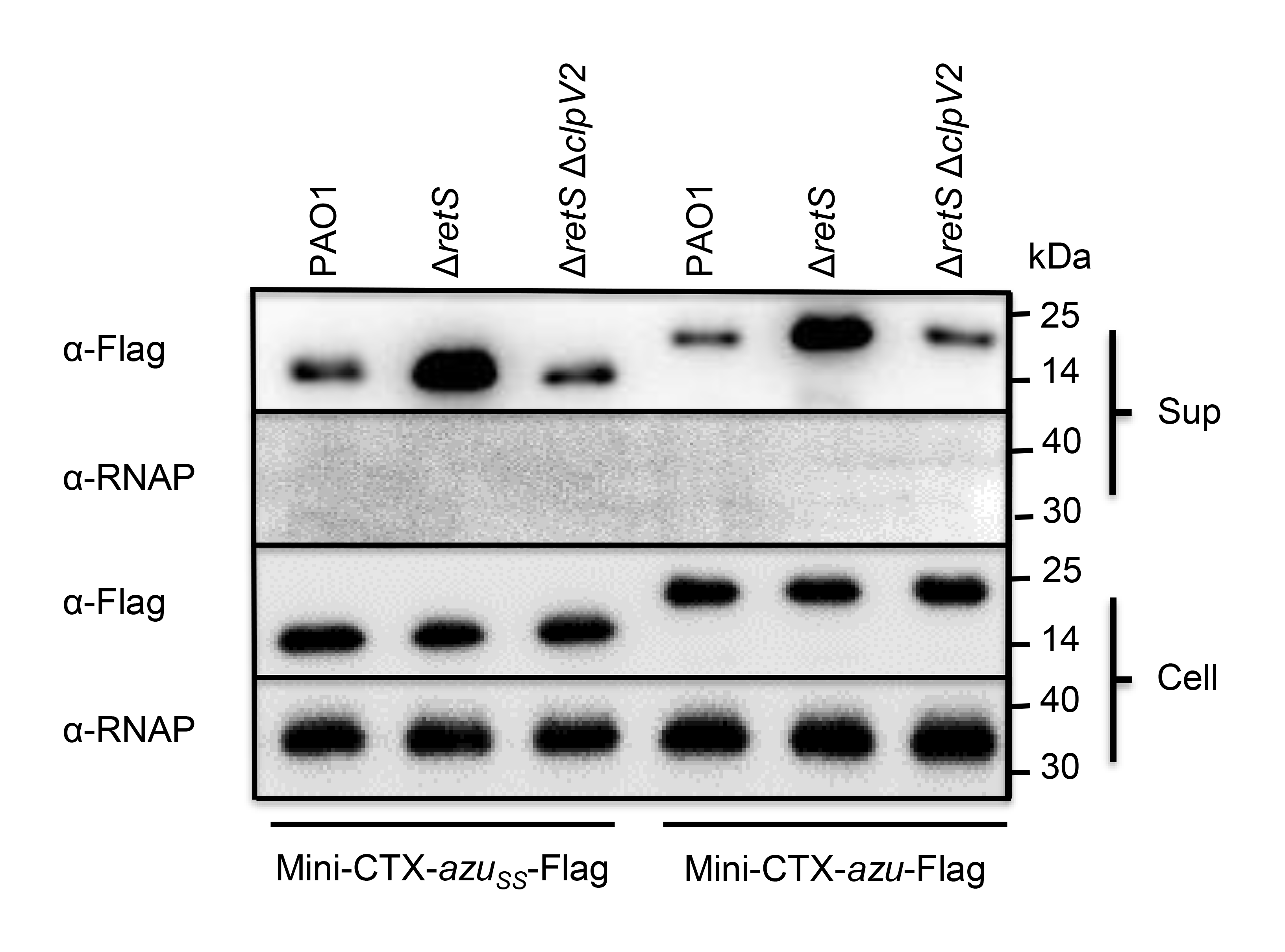

Supplement: S2 Fig — A mini-CTX plasmid directing the expression of wild type azu or lacking the N-terminal signal peptide azu(azuss) were integrated into the P. aeruginosa derivative strains, respectively. Western blot analysis of Azuss-Flag or Azu-Flag in the cell-associated (Cell) and concentrated supernatant (Sup) protein fractions from the indicated strains grown in LB. (TIF) [file ppat.1008198.s006.tif]

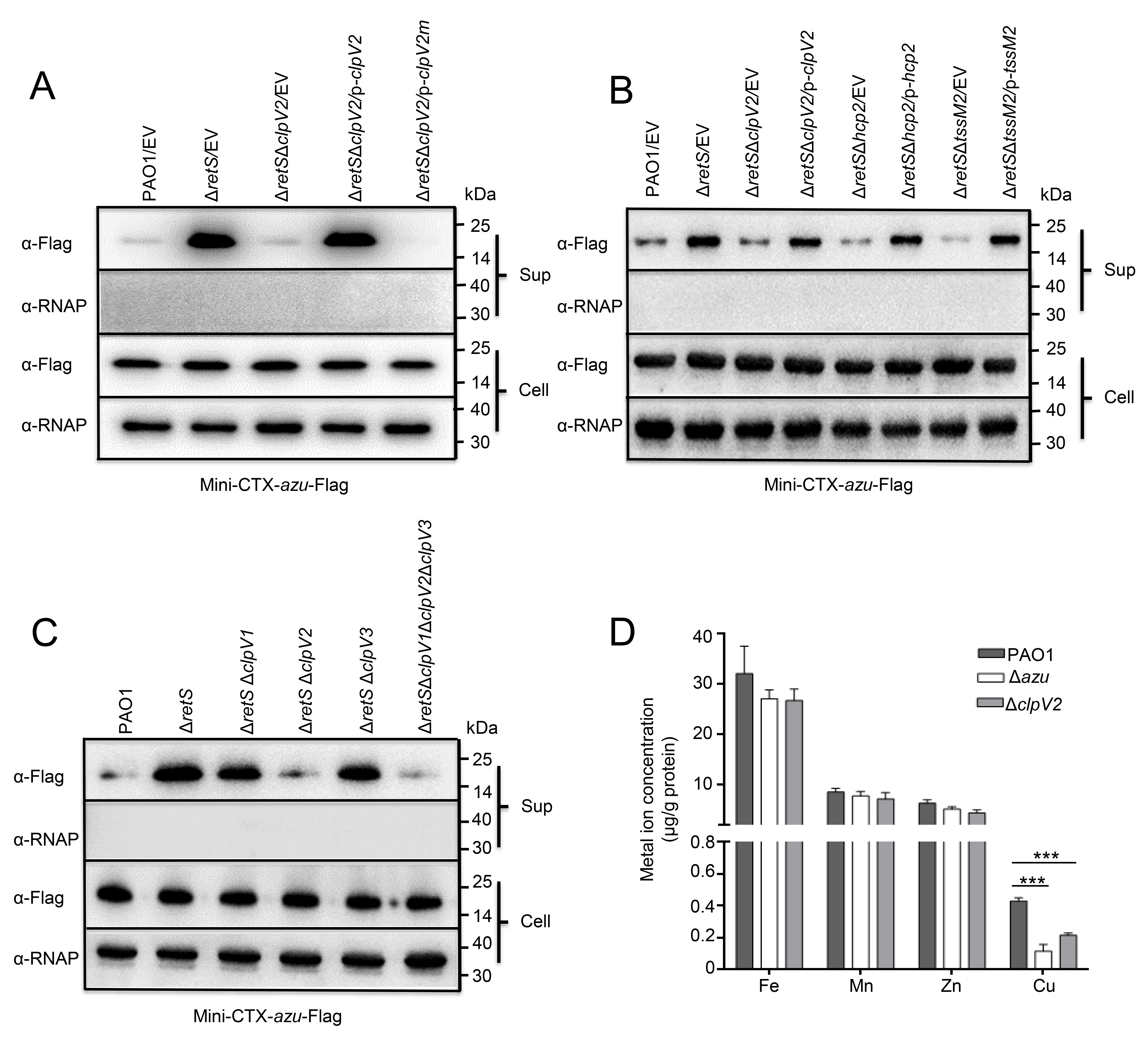

Supplement: S3 Fig — (A) The ClpV2 E286/E692 (ClpV2m) is required for Azu secretion. (B) Secretion of Azu is dependent on Hcp2 and TssM2. (C) H1- and H3-T6SS did not affect the azu activity. (A-C), cell lysates (Cell) and concentrated supernatant (Sup) protein fractions from the indicated strains were separated by SDS/PAGE and proteins were detected by western blot. EV represents the empty vector pAK1900. (D) ICP-MS assays showed that mutation of azu or clpV2 reduced the intracellular Cu2+ levels. Strains were cultured at OD600 = 1.0 in M9 medium containing 1.0 mM EDTA. Cu2+ associated with bacterial cells was measured by ICP-MS. Error bars indicate the mean ± s.d. of three biological replicates, and significance was determined by Student′s t-test: ***P<0.001. (TIF) [file ppat.1008198.s007.tif]

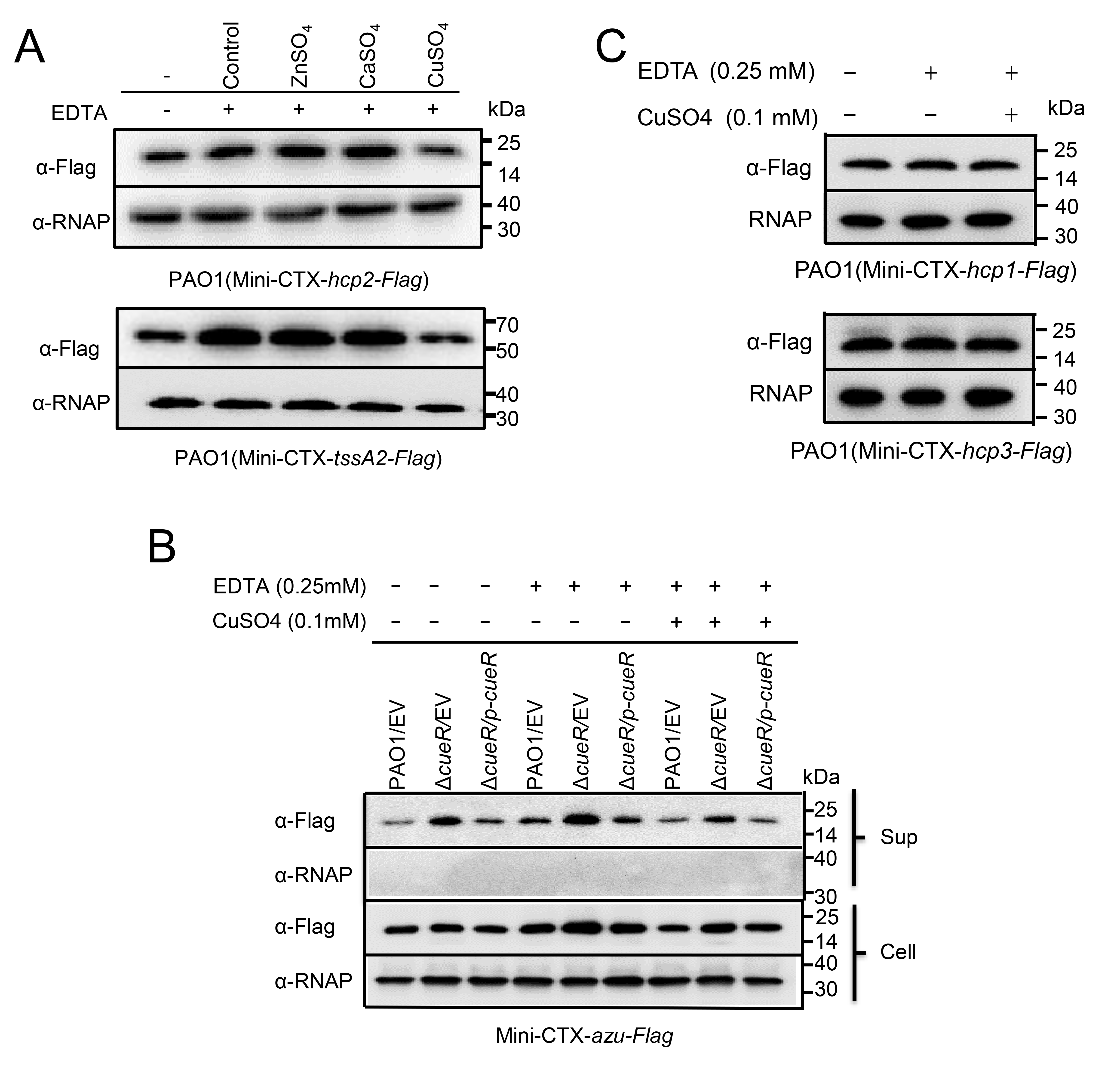

Supplement: S4 Fig — (A) The expression of hcp2 and tssA2 was induced by low Cu2+, but not Zn2+ or Ca2+. A mini-CTX plasmid directing the expression of Hcp2-Flag or TssA2-Flag chimera was integrated into the P. aeruginosa background strain. Bacteria were cultured in LB medium supplemented with either 0.25 mM EDTA or 0.25 mM EDTA with 0.1 mM of ZnSO4, CaSO4 or CuSO4. (B) Western blot analysis showed that 0.25 mM EDTA efficiently activates the azu expression. The activity of Azu was repressed by high Cu2+. (C) The expression of H1- and H3-T6SS was not regulated by Cu2+. (A-C) cell lysates (Cell) and concentrated supernatant (Sup) protein fractions from the indicated strains cultured in LB medium containing EDTA with or without CuSO4 were separated by SDS/PAGE and protein was detected by western blot assays. (TIF) [file ppat.1008198.s008.tif]

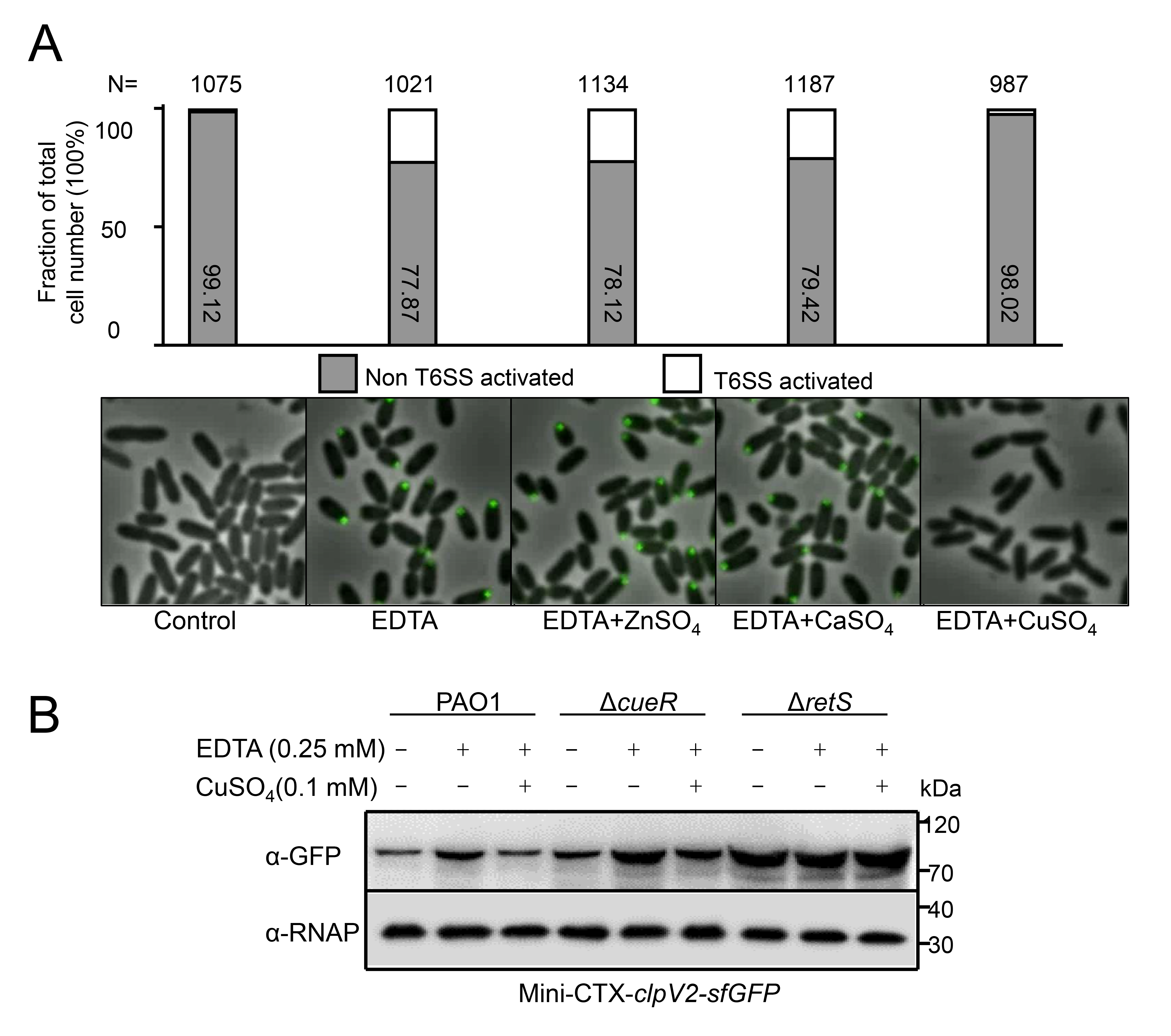

Supplement: S5 Fig — (A) Cu2+ influences H2-T6SS assembly. Chromosomally encoded ClpV2-sfGFP localization in the P. aeruginosa measured by fluorescence microscopy. Cells were grown in the indicated conditions to OD600 = 1.0 and H2-T6SS activated were analyzed. N = total number of cells analyzed for each strain. Bacteria was cultured in LB medium supplemented with either 0.25 mM EDTA or 0.25 mM EDTA with 0.1 mM of ZnSO4, CaSO4 or CuSO4. (B) The stability of ClpV2-sfGFP was not influenced by EDTA, Cu2+, CueR, and RetS. Cell fractions were separated by SDS/PAGE and protein were detected by western blot assays. (TIF) [file ppat.1008198.s009.tif]

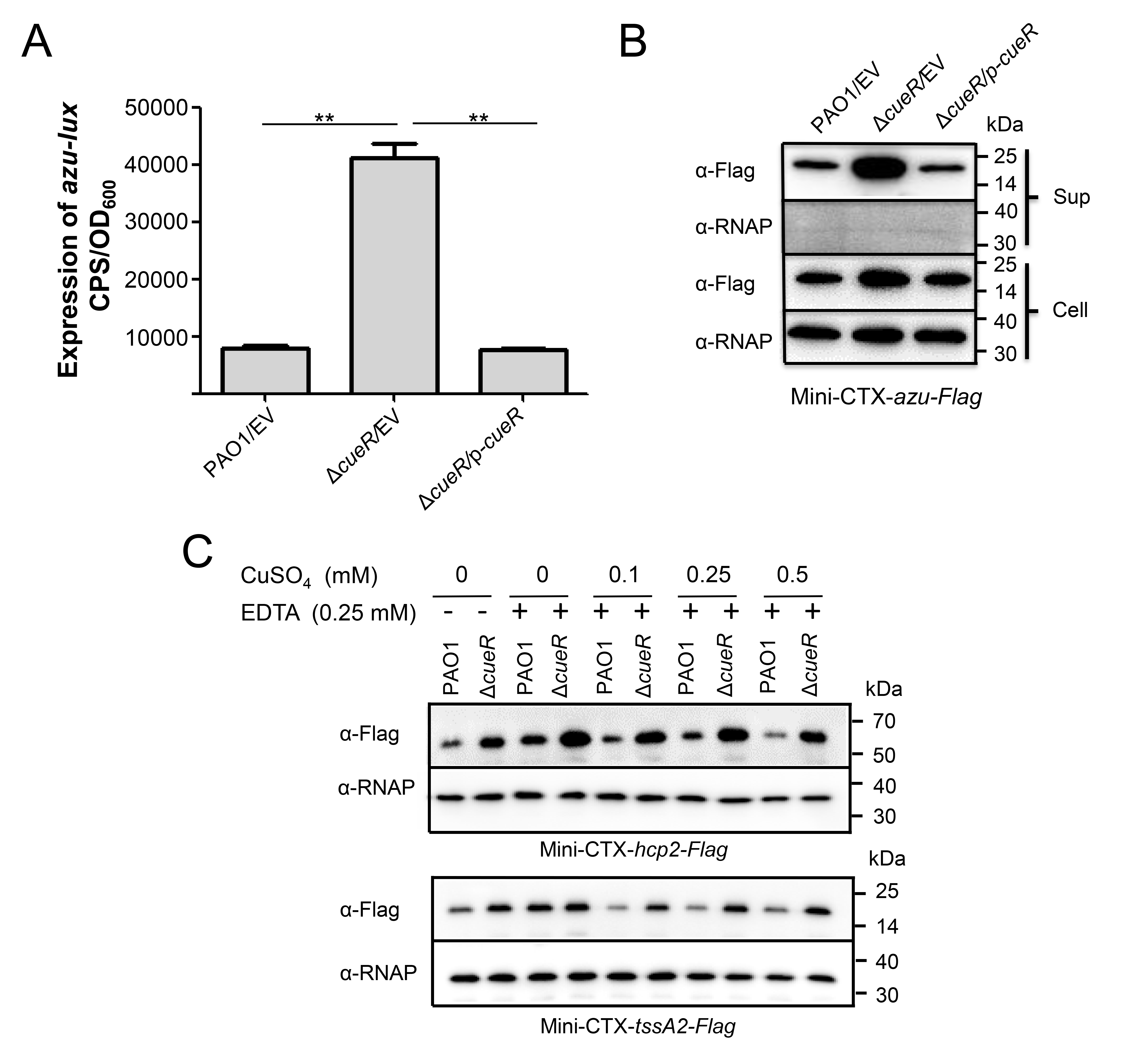

Supplement: S6 Fig — (A) The expression of azu was examined in wild-type PAO1, the cueR mutant, and the ΔcueR complemented strain (ΔcueR/p-cueR). Data shown are the average and SD from three independent experiments. Significance was determined by Student′s t-test, **P<0.01. (B) The indicated strains containing Mini-CTX-azu-Flag plasmid were cultured at OD600 = 1.0 in LB medium. Cell lysates (Cell) and concentrated supernatant (Sup) protein fractions from the indicated strains were separated by SDS/PAGE and proteins were detected by western blot (C) Deletion of cueR increases the levels Hcp2 (upper) and TssA2 (down) relative to wild-type PAO1. Western blot analysis of Hcp2-Flag and TssA2-Flag in the cell-associated fractions from wild-type PAO1 and ΔcueR, strain cultured in LB medium containing 0.25 mM EDTA with or without 0.1, 0.25 and 0.5 mM CuSO4. Cell fractions were separated by SDS/PAGE and protein were detected by western blot assays. (TIF) [file ppat.1008198.s010.tif]

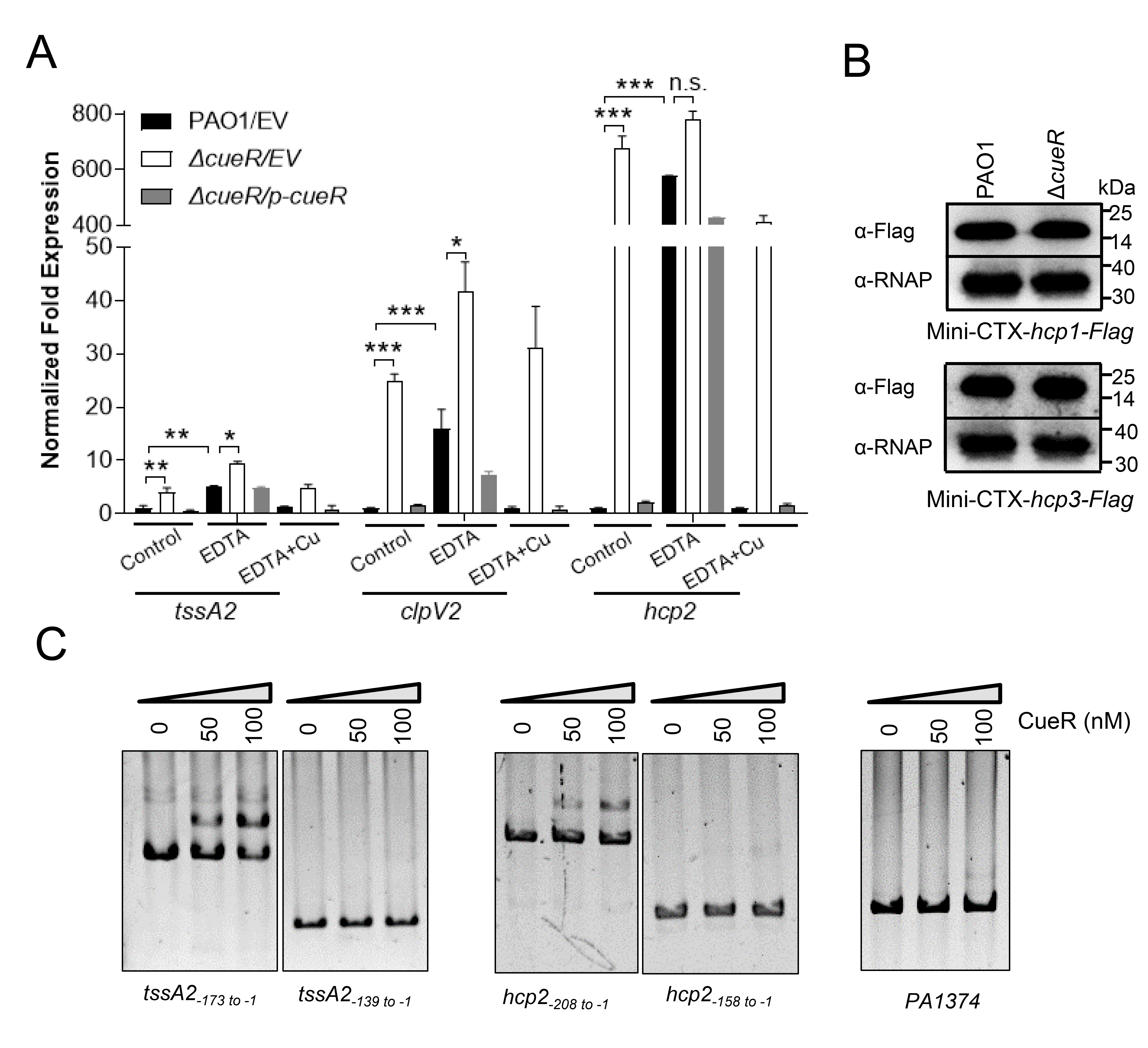

Supplement: S7 Fig — (A) The mRNA levels of tssA2, clpV2, and hcp2 in wild-type PAO1, ΔcueR mutant, and its complemented strain (ΔcueR/p-cueR) was examined by qRT-PCR. Strains were cultured to an OD600 = 0.8 in LB broth or LB supplemented with either 0.25 mM EDTA or 0.25 mM EDTA with 0.1 mM Cu2+.at 37°C. Cultures were harvested, and total RNA was isolated. Error bars represented the mean ± s.d. from three independent experiments. *P<0.05, **P<0.01, ***P<0.001 based on two-way ANOVA Dunnett′s multiple comparison test. NS, not significance. EV represents the empty vector pAK1900. (B) Western blot assays showed that CueR did not influence Hcp1 and Hcp3 protein levels. Intracellular Hcp1 and Hcp3 proteins were separated by centrifugation. Samples were subjected to SDS/PAGE gels and probed with an anti-Flag antibody. (C) EMSAs showed that CueR bound to the truncated fragment of hcp2-208 to -159 and tssA2-173 to -140. PCR products were added to the reaction mixture at a concentration of 2.0 ng. The protein concentration of each sample is indicated above its lane. As a negative control, no band shift was observed when CueR protein incubates with PA1374 promoter region. (TIF) [file ppat.1008198.s011.tif]

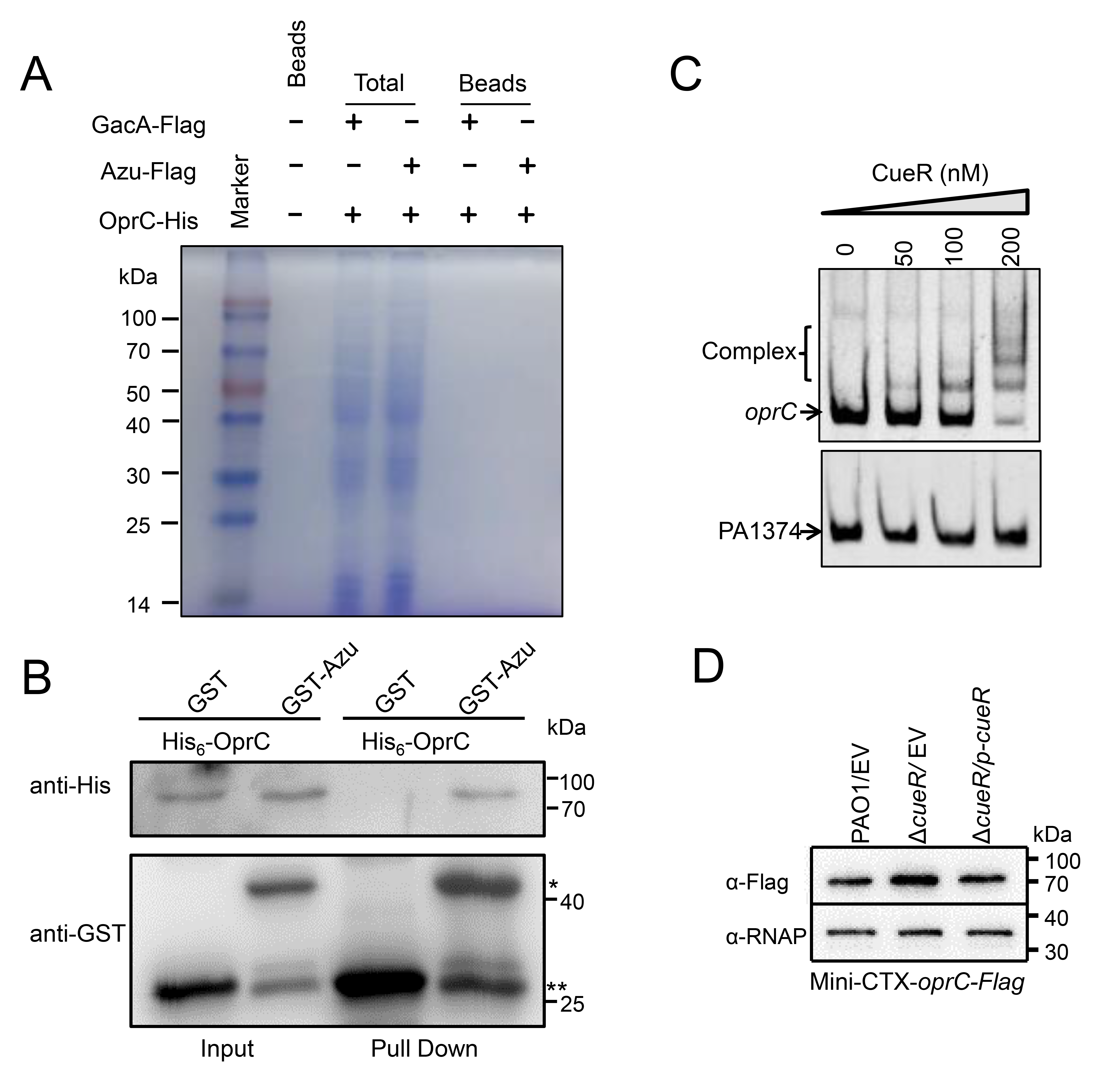

Supplement: S8 Fig — (A) SDS-PAGE analysis of the samples in Co-IP assay. Cell lysates of P. aeruginosa containing pMMB67H-OprC-His with either p-GacA-Flag or p-Azu-Flag were individually incubated with Flag beads, and beads retained proteins were strained by Coomassie Blue R-250. (B) Azu interaction with OprC. His6-OprC was incubated with GST-Azu or GST, and the protein complexes captured with glutathione beads were detected by western blot. The single asterisk and double asterisks represent GST-Azu and GST protein, respectively. Data are representative of two replications. (C) CueR binds to the promoter region of oprC. PCR products were added to the reaction mixtures at 2.0 ng. The PA1374 promoter region showing no binding with CueR protein as a negative control. (D) Protein levels of OprC were tested in wild-type PAO1, ΔcueR mutant, and its complemented strain (ΔcueR/p-cueR). The indicated strains containing OprC-Flag were cultured to an OD600 = 1.0 in LB broth at 37°C. The pellet fractions of cells were analyzed by western blot using anti-Flag antibody. EV represents the empty vector pAK1900. (TIF) [file ppat.1008198.s012.tif]

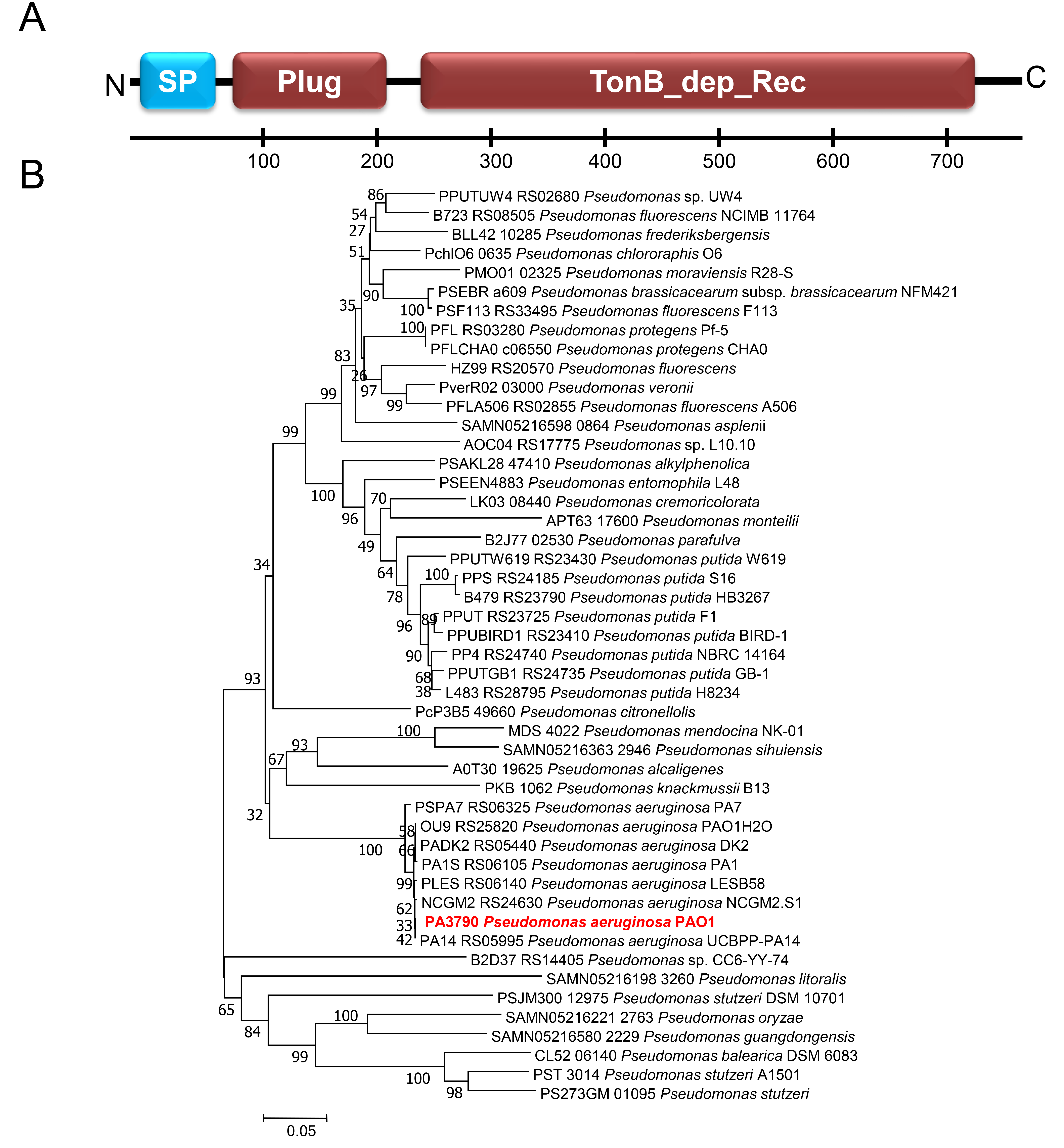

Supplement: S9 Fig — (A) Schematics of conserved domains of OprC identified by using Pfam. Plug, the TonB-plug domain (residues 75–181); TonB-dep-Rec, the TonB-dependent receptor domain (residues 224–722). (B) OprC homologs are widely distributed in Pseudomonas species. The phylogeny generated by the neighbor-joining algorithm in MEGA 7.0 illustrates that OprC is highly conserved among the vast majority of Pseudomonas. The PAO1 OprC was highlighted by red. The bar represents the genetic distance. (TIF) [file ppat.1008198.s013.tif]
